# Chytridiomycosis causes catastrophic organism-wide metabolic dysregulation including profound failure of cellular energy pathways

**DOI:** 10.1038/s41598-018-26427-z

**Published:** 2018-05-29

**Authors:** Laura F. Grogan, Lee F. Skerratt, Lee Berger, Scott D. Cashins, Robert D. Trengove, Joel P. A. Gummer

**Affiliations:** 1 Griffith Wildlife Disease Ecology Group, Environmental Futures Research Institute, School of Environment, Griffith University, Nathan, Queensland 4111 Australia; 2 One Health Research Group, College of Public Health, Medical and Veterinary Sciences, James Cook University, Townsville, Queensland 4811 Australia; 3 Separation Science and Metabolomics Laboratory, Murdoch University, Perth, Western Australia 6150 Australia; 4 Metabolomics Australia, Murdoch University Node, Murdoch University, Perth, Western Australia 6150 Australia

## Abstract

Chytridiomycosis is among several recently emerged fungal diseases of wildlife that have caused decline or extinction of naïve populations. Despite recent advances in understanding pathogenesis, host response to infection remains poorly understood. Here we modelled a total of 162 metabolites across skin and liver tissues of 61 frogs from four populations (three long-exposed and one naïve to the fungus) of the Australian alpine tree frog (*Litoria verreauxii alpina*) throughout a longitudinal exposure experiment involving both infected and negative control individuals. We found that chytridiomycosis dramatically altered the organism-wide metabolism of clinically diseased frogs. Chytridiomycosis caused catastrophic failure of normal homeostatic mechanisms (interruption of biosynthetic and degradation metabolic pathways), and pronounced dysregulation of cellular energy metabolism. Key intermediates of the tricarboxylic acid cycle were markedly depleted, including in particular α-ketoglutarate and glutamate that together constitute a key nutrient pathway for immune processes. This study was the first to apply a non-targeted metabolomics approach to a fungal wildlife disease and specifically to dissect the host-pathogen interface of Bd-infected frogs. The patterns of metabolite accumulation we have identified reveal whole-body metabolic dysfunction induced by a fungal skin infection, and these findings have broad relevance for other fungal diseases.

## Introduction

Recently emerged fungal skin diseases of wildlife include chytridiomycosis in amphibians, white nose syndrome in bats, snake fungal disease and mucormycosis in platypus^1,2^. Knowledge is needed urgently to develop novel approaches for control^3,4^. While targeted metabolite studies have documented host changes associated with white nose syndrome and chytridiomycosis^5,6^, there have been no broad scale metabolomics studies investigating these fungal diseases excepting those aimed at identifying anti-microbial peptides or probiotics^7^. Chytridiomycosis is an often fatal fungal skin disease of amphibians, affecting approximately 42% of species examined^8^ and causing declines and extinctions around the world^9^. Fungal sporangia invade the superficial epidermal cells leading to disrupted skin functions involving ion transport^10^. While the proximate cause of mortality due to the pathogen, *Batrachochytrium dendrobatidis* (hereafter Bd) has been linked with alteration of electrolyte balance (low plasma levels of sodium and potassium) and subsequent cardiac arrest^10^, the accompanying host metabolic phenotype has not been well characterized. Several gene expression studies have identified pathways associated with end-stage physiology. These include increased expression of genes associated with cellular stress and disruption of skin homeostasis, detoxification, proteases, antimicrobial peptides, as well as decreased expression of cytochrome p450 genes, blood coagulation genes and immune genes (although recent evidence suggests the degree and efficacy of immune response may differ between species)^11–18^. An improved understanding of the molecular basis underlying the pathophysiology of chytridiomycosis will be fundamental to improving management techniques and hence population outcomes^3^. In addition, comparing the metabolome of populations with differing long-term exposure histories and susceptibility to Bd may elucidate immune mechanisms associated with more resistant or tolerant phenotypes.

Non-targeted metabolomics (otherwise known as metabonomics or metabolic phenotyping when undertaken to investigate and compare disease states) seeks to measure the small molecule ( <1,500 daltons) component of a biological system. It investigates the precursors, intermediates and products of biochemical pathways (including for example amino acids, organic acids, sugars and nucleotides^19,20^), as well as exogenous influences such as occurring through exposure events. Mass spectrometry (MS) and chemometrics are the cornerstone of modern metabolomics research, capable of discriminating thousands of metabolic features to characterize a metabolic phenotype, or ‘phenome’^21^. Non-targeted metabolomics analyses can identify patterns of metabolite flux to elucidate biochemical perturbations induced by infection and disease^21^. Such insights have led to the development of numerous assays for screening biomarkers of disease, particularly in humans^22–24^. Metabolomics approaches have also been widely used in toxicological and pharmacological studies^7,25,26^.

To date, numerous studies have used mass spectrometry to characterize the interaction between Bd and the amphibian host including investigations of intra and extracellular fungal metabolites, amphibian urinary metabolic markers of stress, epidermally secreted antimicrobial peptides, and the analysis of antifungal metabolites produced by symbiotic skin bacteria^7,27–32^. These studies have greatly furthered our understanding of frog-fungal pathosystems. Thus far, however, no study has attempted to examine the overall metabolic phenotype (including intracellular and within multiple cell types and organs) associated with chytridiomycosis throughout pathogenesis. Moreover, no study has yet examined differences in metabolic phenotype among different populations of a single frog species with varying exposure histories and clinically demonstrated differential susceptibility to disease.

In this study we used a non-targeted metabolomics approach to examine the longitudinal metabolic phenotype of chytridiomycosis infection post exposure, as well as to compare the metabolome of different populations of the same species with differing long-term Bd-exposure histories and survival responses. We analyzed skin tissues at the site of infection, as well as liver tissues from exposed and unexposed control frogs at multiple time points throughout disease progression (subclinically at four, eight and 14 days post exposure, and in clinically diseased animals).

## Materials and Methods

### Sample collection

#### Ethics Statement

Wild alpine tree frog eggs (*Litoria verreauxii alpina*) were collected by D. Hunter in accordance with Scientific License number: S12848. Animals were captively-raised by experienced animal handlers under quarantine conditions following Taronga Conservation Society Animal Ethics Committee guidelines (4c/01/10). Experimental protocols involving animals were similarly carried out in accordance with the approved guidelines and protocols under permits issued by James Cook University (A1408) and Taronga Conservation Society (4c/01/10) Animal Ethics Committees. Animals were individually examined at least once daily for clinical signs of chytridiomycosis infection (lethargy, peripheral erythema and increased skin shedding), and humanely euthanized if clinical signs were detected (typically indicative of end-stage disease)^33,34^.

#### Study subjects, exposure experiment and sampling

Source populations, study subjects, exposure experiment and sampling protocols are described in detail by Grogan *et al*.^33^ (refer to Experiment B). In brief, 61 chytridiomycosis-naïve adult alpine tree frogs were captively-raised from wild-caught eggs collected from four geographically distinct populations in Kosciuszko National Park, New South Wales, Australia. *Batrachochytrium dendrobatidis* had emerged at three of these sites (hereafter Kiandra, Eucumbene and Ogilvies) over two decades earlier than the time of egg collection (as determined via long-term monitoring^33^). The remaining site (hereafter Grey Mare) was known to be Bd-naïve at the time of egg collection. Forty-six of these frogs were individually exposed to 750,000 zoospores of Bd using the AbercrombieNP-L.booroolongensis-09-LB-P7 isolate in a topical bath of dilute salts solution (DSS)^33^, while the remaining 15 frogs were sham-exposed with only DSS (unexposed negative control group). Seventeen animals (including both exposed and control animals across populations) at each of three subclinical sampling sessions (at either 4, 8 or 14 days post-exposure) were skin-swabbed to quantify Bd infection intensity, euthanized and then had skin and liver tissues sampled. The remaining 10 animals were swabbed, euthanized and sampled at the onset of clinical signs (between 28 and 30 days post-exposure; see Table 1 for details of experimental design). Ventral abdominal skin (common site of infection) and liver tissues were collected into 500 µL 100% methanol and stored at −80 °C.

**Table 1:** Experimental design outlining the number of frogs from each population and treatment group (Bd exposed or unexposed control) sampled at each time point post exposure.

Populations	Exposure – Day 0 Total # exposed (total # control)^a^	Day 4 # exposed sampled (# control sampled)^b^	Day 8 # exposed sampled (# control sampled)^b^	Day 14 # exposed sampled (# control sampled)^b^	Clinically diseased (Day 28+) # exposed sampled
Grey Mare	16 (3)	4 (1)	4 (1)	4 (1)	4
Eucumbene	14 (6)	4 (2)	4 (2)	4 (2)	2
Kiandra	14 (6)	4 (2)	4 (2)	4 (2)	2
Ogilvies	2	—	—	—	2

^a^Total number of unexposed control frogs shown in parentheses; ^b^Number of unexposed control frogs sampled shown in parentheses.

### Metabolomics methods

#### Isolation, preparation and GC-MS analysis

Details of the metabolomics preparative methods are provided by Grogan *et al*.^33^. In brief, harvested liver and skin tissues were dried by lyophilisation, homogenized via agitation, and metabolites extracted into a methanol supernatant via centrifugation, before being dried via vacuum concentration and lyophilisation. An additional pooled extract was created for follow-up analyses. The metabolites were then trimethylsilyl (TMS) derivatised in preparation for gas chromatography mass spectrometry (GC-MS) as previously described^33^. Five µL of hexane containing a series of straight-chain alkanes was added to samples for the calculation of retention indices (RIs). The GC-MS (Shimadzu QP2010 Ultra, Kyoto, Japan) analysis of the metabolite derivatives was described by Grogan *et al*.^33^, with data acquired and analysed using GCMSsolution 2.61 (Shimadzu Corporation, Kyoto, Japan) and AnalyzerPro 2.7.0.0 (Spectral Works, Runcorn, UK). A preliminary scan acquisition (m/z 50–650) was followed by a simultaneous full scan and selective ion monitoring (SIM) mode of acquisition. Gas Chromatography Mass Spectrometry analyses of skin samples were not obtained for seven frogs (five from Kiandra, one from Eucumbene and one from Grey Mare) due to a precipitate that formed in the derivatised samples (we could not identify the cause for the precipitate). All analytes were relatively quantified using a characteristic quantifier ion selected for each analyte, and metabolite identities assigned by comparison to authentic metabolite standards or by reference to an external mass spectral library (Massbank, National Institute of Standards and Technology (NIST) or Wiley Registry™). Metabolites without a match were labelled with the prefix ‘unknown’, followed by the observed retention time (RT) and calculated RI. Quality control and technical validation procedures are described in detail by Grogan *et al*.^33^.

### Data analysis and interpretation

Measured analytes of non-biological origin were determined by comparison to no-tissue control preparations, which were prepared identically to the sample extracts, but without skin or liver tissue. Any non-biological analytes were subsequently removed from the data matrices. Peak areas were normalized to the summed total ion intensity^35^, subsequently log transformed^36^ (log_10_(x + 1)) and pareto-scaled (mean-centered values are divided by square root of the standard deviation^37^). Statistical analyses were performed with SPSS (IBM, USA), R (Bell Laboratories), and Metaboanalyst 3.6^38^. For ease of interpretation, data from sham-exposed negative control group frogs (for all analyses except batch effect analyses) were designated Group 0, regardless of whether they were sampled at 4, 8 or 14 days post-exposure. Exposed/infected frogs sampled at various times post-exposure were designated Groups 1, 2, 3 and 4, corresponding to being sampled at 4, 8, 14 and 28–30 days post exposure, respectively. The numbers of samples included in each group for each analysis have been described below, and varied depending on original sample size and taking into account any missing values.

Traditional univariate statistical analyses included Analysis of Variance with post-hoc Tukey’s Honestly Significant Difference (HSD) tests, a pattern matching correlation analysis with Pearson r correlation^39^, and Significance Analysis of Microarrays (SAM^40^). In all cases, an alpha error level of 0.05 corrected for multiple testing via Bonferroni method was considered acceptable (corresponding to a false discovery rate, FDR < 0.05). Multivariate analyses included unsupervised Principal Components Analysis (PCA) and supervised Partial Least Squares Discriminant Analysis (PLS-DA) providing Variable Importance in Projection scores (VIP) with model quality assessed by 10-fold cross-validation based on Q^2^ and goodness of fit evaluated with permutation testing using 2,000 iterations. We also used the Omics Dashboard^41^ to explore patterns of metabolite accumulation and depletion across metabolic pathways and cellular systems in response to chytridiomycosis. Population comparisons excluded data on the two frogs from Ogilvies due to small sample size, however these were included in the sample period based analysis. Metabolites found most influential within the data modelling were searched for in intracellular and extracellular metabolite analyses of Bd sporangia and culture medium (data not shown), to determine if of frog or fungal origin.

## Results

### Clinical characteristics and summary results

All 61 experimental animals survived the duration of the experiment until they were euthanized for sampling. As expected, the 10 frogs sampled between 28 and 30 days post-exposure were demonstrating clinical signs of chytridiomycosis (muscle weakness, lethargy, peripheral erythema or inability to maintain normal upright posture) at the time of euthanasia. Demographic data on the sampled frogs, together with infection intensities as assessed via qPCR can be found in Table 2. Data from a concurrent large survival experiment using other animals from these same populations demonstrated that frogs from Kiandra survived significantly longer when compared with frogs from the other populations^33,42,43^.

**Table 2:** Demographic characteristics of study subjects (including sample size, treatment group, gender ratios, mean mass at death, mean snout-urostyle length at death and mean and median infection intensity at death).

	Kiandra		Eucumbene		Grey Mare		Ogilvies	P value^a^	df^a^
	Exposed	Control	Exposed	Control	Exposed	Control	Exposed		
Sample size	14	6	14	6	16	3	2		
Gender^b^	3M, 4F, 7U	2M, 4U	3M, 6F, 5U	1M, 5F	5M, 7F, 4U	2F, 1U	1M, 1F		
Mean mass at death	3.21	3.02	2.95	4.05	3.43	3.19	3.29	0.917	3
Mean SUL at death^c^	30.29	30.72	28.79	32.10	32.01	32.03	31.80	0.150	3
Mean ZSE at death^d^	429122.80	0.28	145852.98	0.00	148017.50	0.00	614937.50	0.697	3
Median ZSE at death	705.83	0.00	185.83	0.00	5955.83	0.00	614937.50		

^a^Statistics comparing means between populations (pooling exposed and control frog values) using one-way ANOVA, P value and df degrees of freedom; ^b^Genders represented by M for males, F for females and U for unknown gender; ^c^SUL is snout-urostyle length measured with Vernier callipers; ^d^ZSE is zoospore equivalents as measured by qPCR.

Overall, 23,868 MS features were resolved by GC-MS across both liver and skin tissues with a total of 2,177 zero values and no missing values. The MS features were deconvoluted into peaks representing individual metabolites and the relative quantitation of 162 metabolites was obtained, with 72 of these metabolites identified or putatively identified. Complete metabolite accumulation data have been archived at the MetaboLights data repository and Dryad Data Repository (details in Grogan *et al*.^33^). While we refer to acid analytes by their library standard name in the results, in the discussion we instead refer to the equivalent anion salt with the suffix ‘-ate’ (ie, citric acid becomes citrate), as this is the biologically relevant form.

The effect of sample processing was identified to be minimal via a batch effect PCA analysis, consistent with preventative GC-MS maintenance regimens (see Supplementary Fig. S1 and Grogan *et al*.^33^). It was also clear from this analysis that the metabolite profiles of skin tissue samples clustered separately from liver samples, as expected with differential underlying tissue physiology. Skin and liver samples from the frog designated Lva259 from the Grey Mare population were consistently found to be marked outliers in the PCA results (across analyses; see Supplementary Fig. S1), and hence were removed from further analysis. These two samples may have been outliers due to tissue autolysis post mortem that may have altered the metabolic profile relative to other samples (this individual died from chytridiomycosis in the short interval between swabbing and euthanasia).

### Sampling period comparisons

#### Univariate analyses

**Figure 1:**
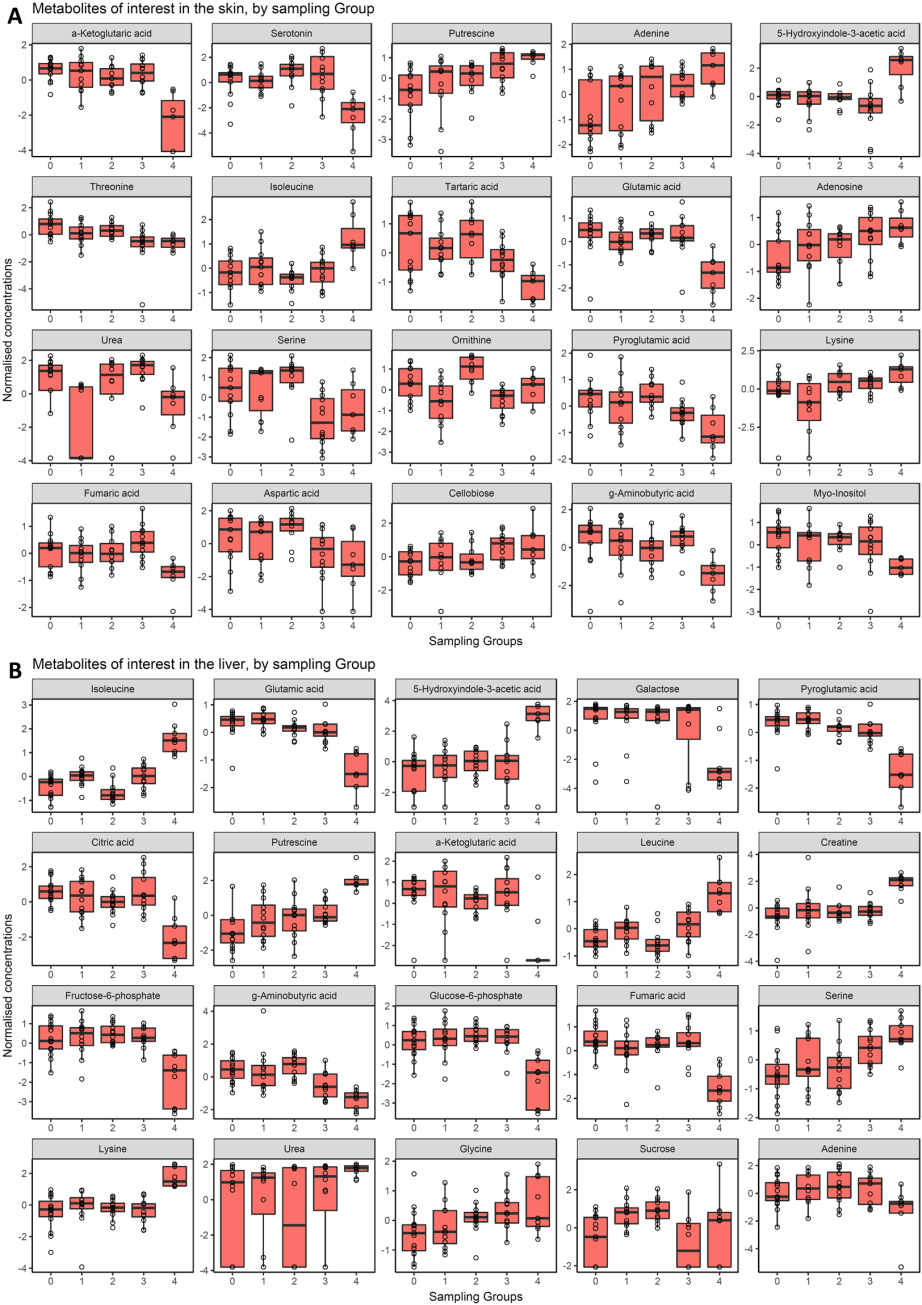
Box and whisker plots (with data points overlaid) of the relative concentration of identified metabolites of interest according to their sampling group (using geom_boxplot from ggplot2 R package). (**A**) Metabolites from skin samples, (**B**) metabolites from liver samples. Sham-exposed negative control group frogs were designated Group 0. Exposed/infected frogs sampled at various times post-exposure were designated Groups 1, 2, 3 and 4, corresponding to being sampled at 4, 8, 14 and 28–30 days post exposure, respectively. Box hinges represent first and third quartiles, and middle represents the median. Whiskers extend 1.5 times the inter-quartile range of the hinge. Boxes are ordered by relative metabolite importance.

One-way ANOVA of **skin** samples identified 33 metabolites (7 unidentified) as differing significantly in relative concentration between **sampling periods** (for this analysis, Groups 0–4 contained 13, 11, 10, 12 and 7 samples respectively). Tukey’s HSD post-hoc tests identified the 10 most significantly differing (FDR < 0.05) metabolites between sampling groups to include α-ketoglutaric acid, serotonin, 5-hydroxyindole-3-acetic acid (5HIAA), isoleucine, glutamic acid, ornithine, tartaric acid, urea, threonine, and serine (Supplementary Table S1). Most of these were associated with lower concentrations in the clinically diseased group of frogs (Group 4) compared with both negative control and subclinical groups (Groups 0–3), except for 5HIAA and isoleucine (where Group 4 was relatively higher), urea (where Groups 1 and 4 were low) and lysine (where Group 1 was low) (Fig. 1A). The metabolites differing significantly in relative concentration between Groups *excluding* the clinically diseased group (ie, only among Groups 0–3) included ornithine, urea, threonine, serine, adenine, putrescine and lysine (Supplementary Table S1). The t-test pattern matching approach using a time-series pattern (Groups 0–4 in order) yielded some differences to the above list. The 10 most significantly differing metabolites identified included α-ketoglutaric acid, putrescine, adenine, threonine, tartaric acid, adenosine, glutamic acid, pyroglutamic acid, cellobiose and myo-inositol (Supplementary Table S2). The SAM analysis identified the following top 10 metabolites as significantly different in concentration between sampling periods: α-ketoglutaric acid, serotonin, 5HIAA, urea, glutamic acid, serine, threonine, ornithine, isoleucine, and lysine (Supplementary Table S3).

**Liver** samples grouped by **sampling period** and analyzed with one-way ANOVA revealed 79 metabolites as differing significantly in relative concentration, 43 of which were positively identified (Groups 0–4 contained 15, 12, 12, 12 and 9 samples respectively). The top 10 identified and most significantly differing metabolites via Tukey’s HSD post-hoc tests included isoleucine, glutamic acid, pyroglutamic acid, leucine, putrescine, fructose-6-phosphate, citric acid, fumaric acid, glucose-6-phosphate and γ-aminobutyric acid (GABA; Supplementary Table S4). Once again, the majority of these were associated with lower concentrations in the clinically diseased group of frogs (Group 4) compared with the remaining sample Groups (Groups 0–3), except for isoleucine, leucine and putrescine which were all elevated in Group 4 (Fig. 1B). Top identifiable metabolites that differed significantly between subclinical and control groups included isoleucine, leucine, GABA, serine, sucrose and cellobiose (Supplementary Table S4). Searching for patterns matching the time-series of sampling periods (similar to above) yielded the following top 10 identifiable metabolites as being highly significant: glutamic acid, pyroglutamic acid, putrescine, isoleucine, leucine, GABA, serine, creatine, citric acid and fumaric acid (Supplementary Table S5). The SAM analysis presented different rankings, including the following as the 10 most significant identifiable metabolites: 5HIAA, citric acid, putrescine, α-ketoglutaric acid, galactose, fructose-6-phosphate, creatine, glucose-6-phosphate, isoleucine and lysine (Supplementary Table S6).

#### Multivariate analyses

**Figure 2:**
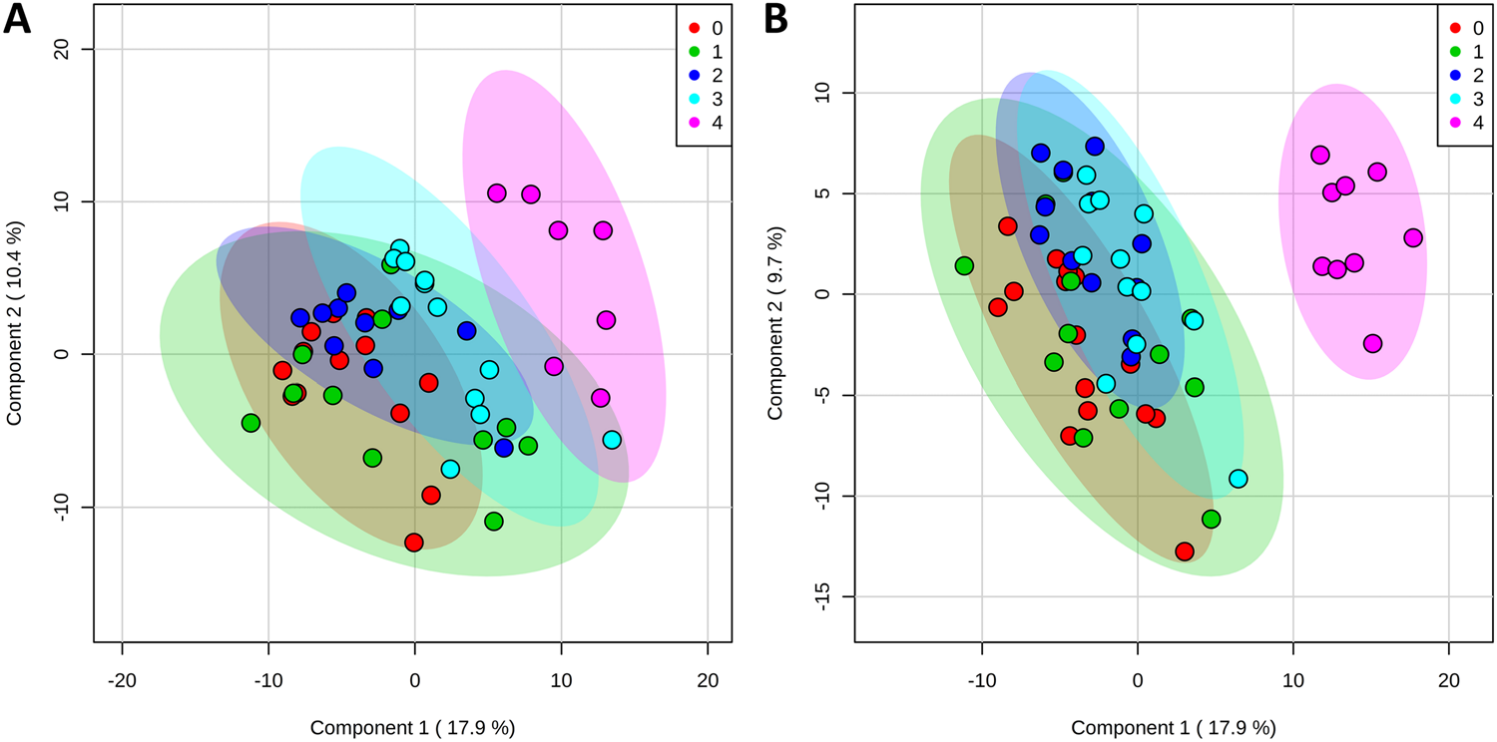
Partial Least Squares discriminant analysis (PLS-DA) scores plots of components one and two, comparing skin samples (**A**) and liver samples (**B**) as they cluster by sampling group. Sham-exposed negative control group frogs were designated Group 0. Exposed/infected frogs sampled at various times post-exposure were designated Groups 1, 2, 3 and 4, corresponding to being sampled at 4, 8, 14 and 28–30 days post exposure, respectively.

Supervised partial least squares discriminant analysis (PLS-DA) demonstrated marked separation between clinically diseased frog tissues (Group 4) and control and subclinically infected tissues (Groups 0–3), an effect most pronounced within the liver tissue samples (Fig. 2). The top 30 metabolites ranked by VIP scores based on component one for **skin** samples grouped by **sampling period** revealed the following identified metabolites: α-ketoglutaric acid, putrescine, adenine, threonine, serine, lysine, aspartic acid, serotonin, and cellobiose (Supplementary Table S7, Fig. 1A). Variable importance in projection scores for **liver** samples grouped by **sampling period** yielded the following metabolites as most important ranked by component one: galactose, 5HIAA, putrescine, citric acid, creatine, α-ketoglutaric acid, fructose-6-phosphate, GABA, lysine, isoleucine, urea, glutamic acid, and pyroglutamic acid (Supplementary Table S8, Fig. 1B).

#### Pathway analyses

**Figure 3:**
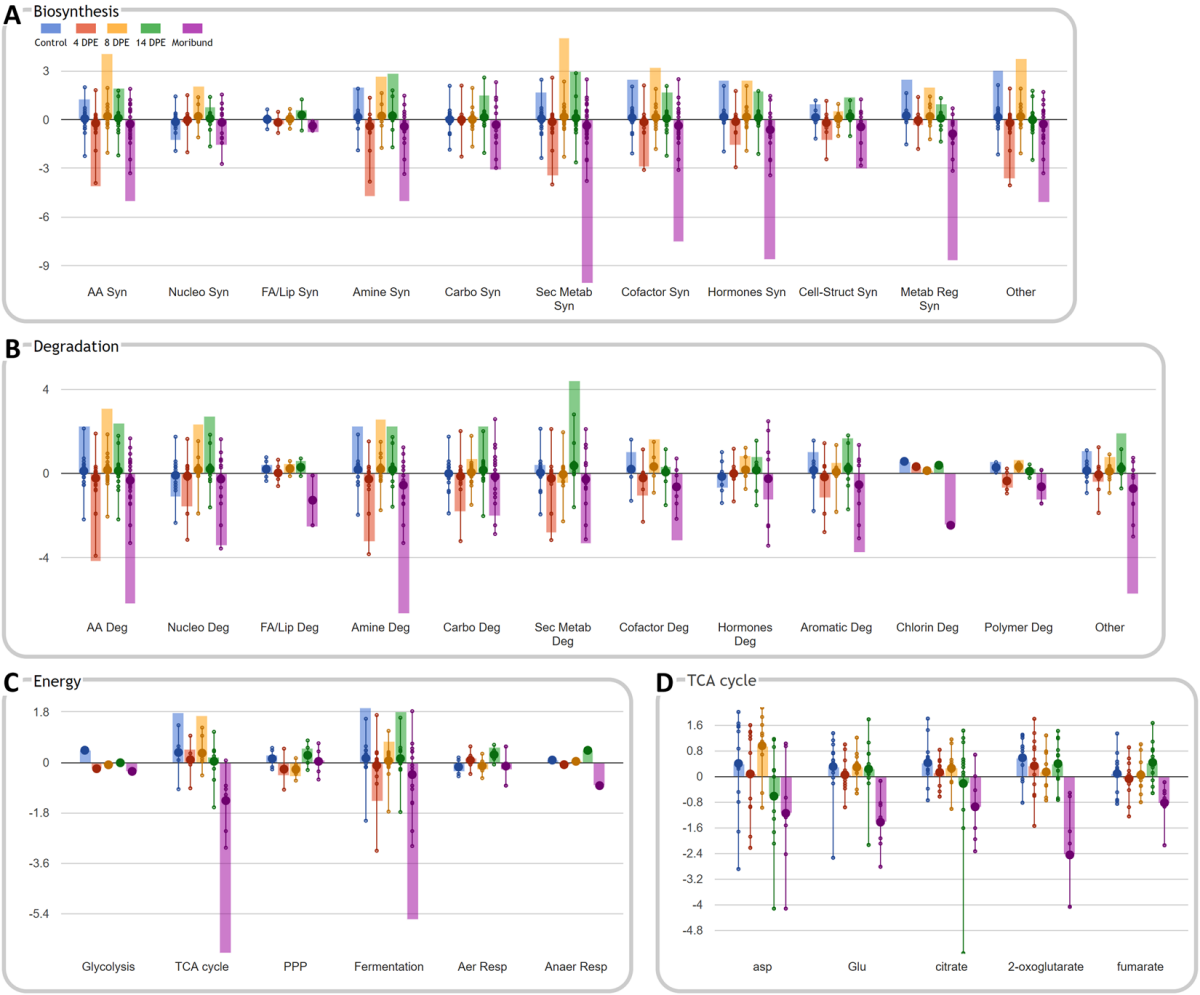
Relative accumulation levels of skin metabolites (from frogs grouped by sampling period) associated with key cellular systems (**A**) Biosynthesis, (**B**) Degradation, (**C**) Energy, and (**D**) relative accumulation data for tricarboxylic acid cycle subsystem intermediates. Large dots represent mean data values for relevant metabolites involved in various subsystems, and small dots represent individual metabolite values. Vertical axis represents normalized relative metabolite accumulation values. Solid colour bars provide the cumulative sum of data values for relevant metabolites, and are more pronounced where many metabolites make a consistent contribution to change within a subsystem.

**Figure 4:**
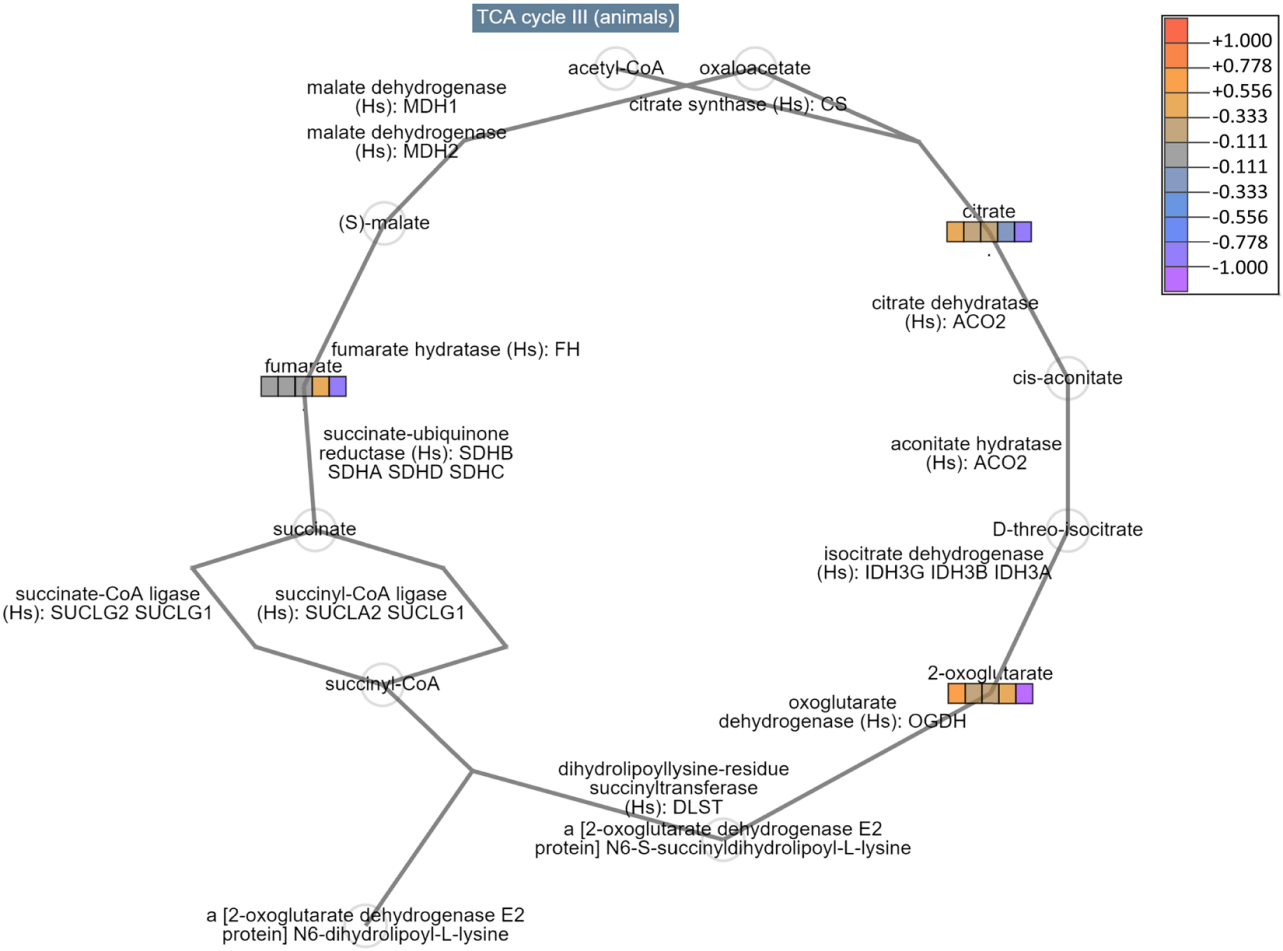
Schematic representation of key intermediates and enzymes involved in the tricarboxylic acid (TCA) cycle. The relative concentration of three key metabolites (citrate, α-ketoglutarate [2-oxoglutarate] and fumarate) is illustrated with the coloured boxes, representing the respective frog sample groups from left to right: (i) negative controls, (ii) 4 days post exposure (DPE), (iii) 8 DPE, (iv) 14 DPE, and (v) moribund.

Interactive exploration (Omics Dashboard^41^) of skin and liver tissue metabolite accumulation data grouped by sampling period revealed substantial alterations in cellular system profiles in clinically diseased frogs. Most notably, numerous metabolites associated with biosynthetic, degradation and energy pathways were depleted in moribund frogs (Group 4) compared with control and subclinically infected frogs (Groups 0–3; see Fig. 3). Nucleotide, carbohydrate, secondary metabolism, cofactor and hormone pathways were markedly affected in both skin and liver tissues of moribund animals (biosynthesis and degradation decreased). Metabolites associated with metabolic regulation synthesis were also depleted in both tissues. Amino acid and amine biosynthetic and degradation pathways were particularly decreased in the skin of moribund animals. Metabolites associated with detoxification pathways were also depleted in both skin and liver tissues of moribund frogs. Metabolites associated with the tricarboxylic acid (TCA, Krebs or citric acid) cycle that were depleted in tissues of moribund frogs included α-ketoglutaric, glutamic, aspartic, citric and fumaric acids (Figs 3 and 4). Low concentrations of citric, fumaric, α-ketoglutaric, and glutamic acids, as well as fructose-6-phosphate, beta-alanine, glycine, hypoxanthine and guanine suggested reduction in the function of energy generating fermentation pathways including degradation of purine nucleobases and fermentation of pyruvate. These results are consistent with the traditional statistical analyses, but build a more comprehensive picture of the effect of chytridiomycosis on respective systems and subsystems.

### Population comparisons

#### Univariate analyses

**Figure 5:**
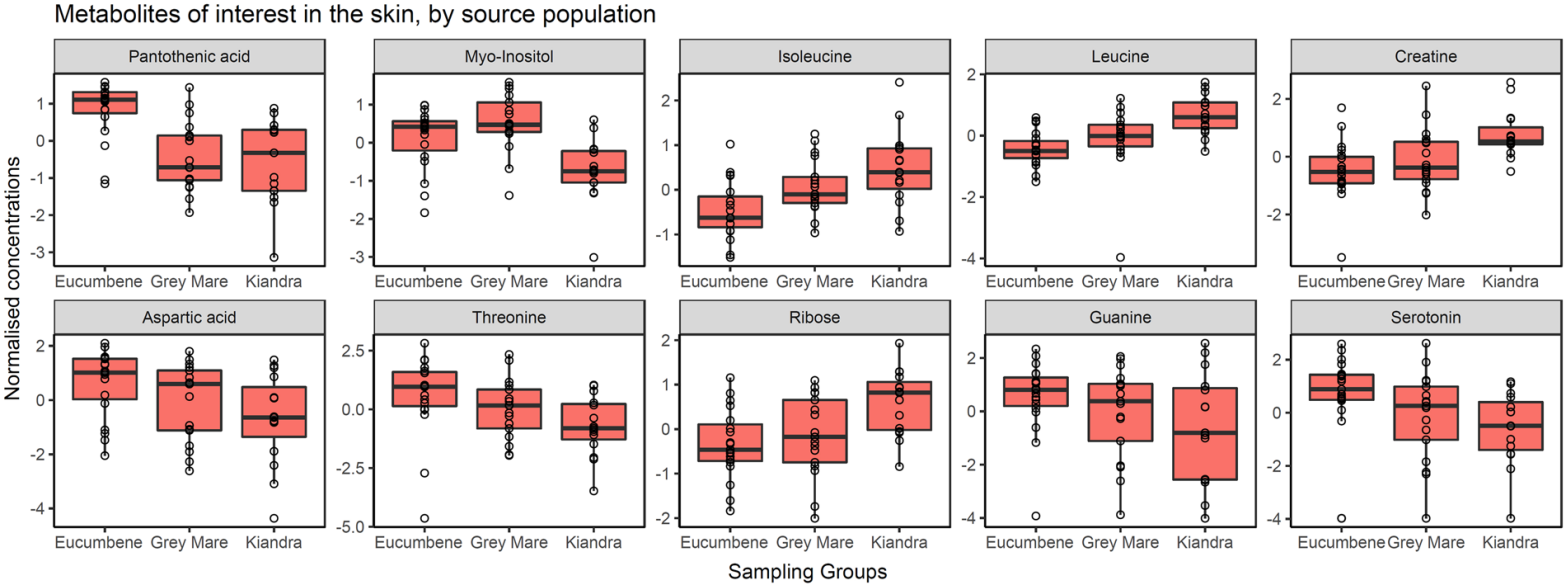
Box and whisker plots (with data points overlaid) of the relative concentration of identified metabolites of interest from skin samples according to their population of origin (using geom_boxplot from ggplot2 R package). Box hinges represent first and third quartiles, and middle represents the median. Whiskers extend 1.5 times the inter-quartile range of the hinge. Boxes are ordered by relative metabolite importance.

One-way ANOVA of **skin** samples grouped according to **population** revealed 11 metabolites differing significantly in relative concentration between populations based on Tukey’s HSD post-hoc tests, 6 of which were unable to be matched to our libraries (populations Eucumbene, Grey Mare and Kiandra contained 19, 17 and 15 samples respectively). Those metabolites identified included pantothenic acid, myo-inositol, leucine, isoleucine and creatine (Fig. 5; Supplementary Table S9). The t-test template matching approach with the pattern Eucumbene-Grey Mare-Kiandra (described below) yielded the following significant metabolites: pantothenic acid, isoleucine, leucine, creatine and ribose (Supplementary Table S10). SAM results identified pantothenic acid, myo-inositol, creatine and leucine as significantly different between populations (Supplementary Table S11). There were no metabolites from **liver** samples identified with any significantly differing relative concentrations between populations with FDR < 0.05 (sample sizes for these analyses included Eucumbene, Grey Mare and Kiandra containing 20, 18 and 20 samples respectively).

#### Multivariate analyses

**Figure 6:**
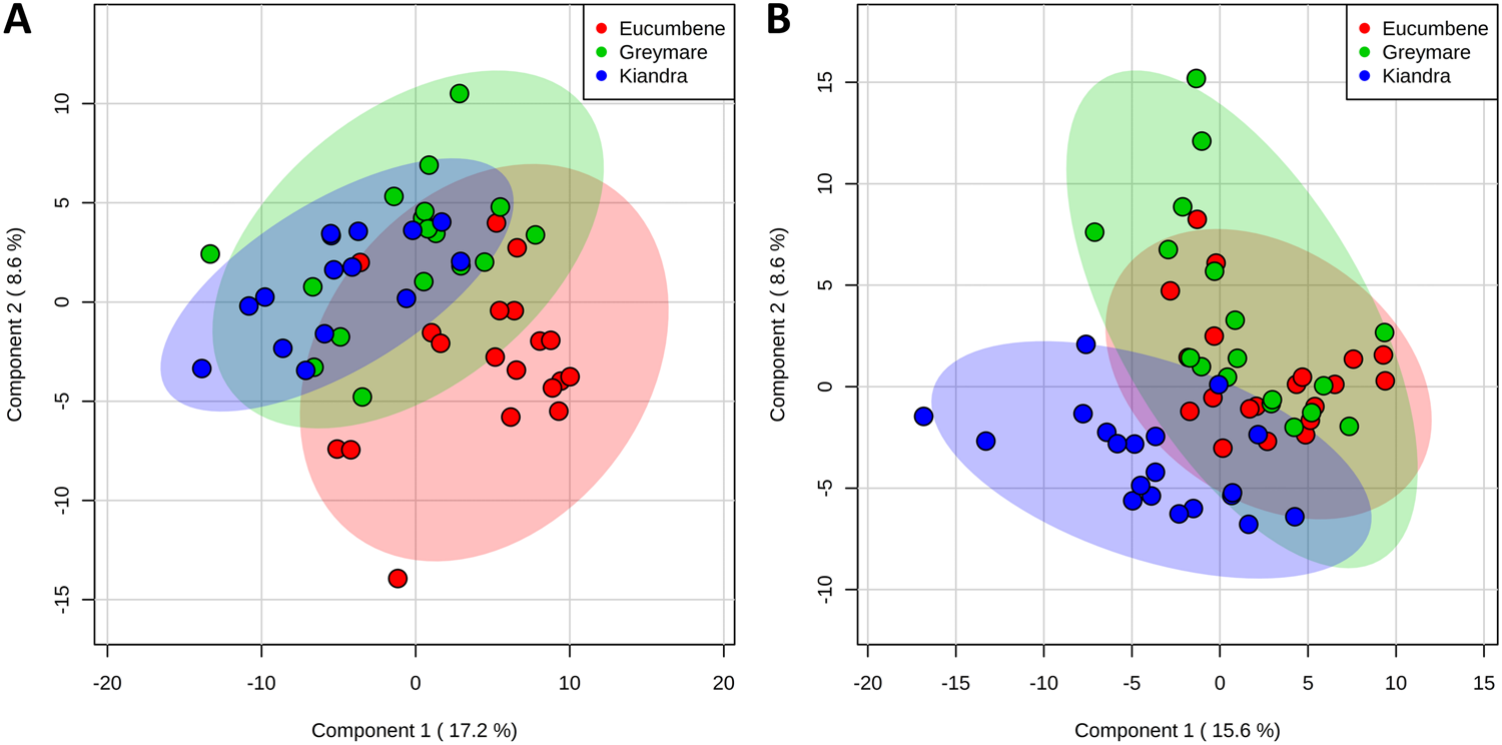
Partial Least Squares discriminant analysis (PLS-DA) scores plots of components one and two, comparing skin samples (**A**) and liver samples (**B**) as they cluster by source population.

Minimal separation of **population** clusters was apparent with supervised PLS-DA for both skin and liver tissue samples. Clustering was more pronounced with the skin samples, where samples from Grey Mare (Bd-naïve population) projected between clusters of samples from the two long Bd-exposed populations (Kiandra and Eucumbene) (Fig. 6). Top ranked identified metabolites contributing to this mild effect for **skin** samples grouped by population using VIP scores included pantothenic acid, aspartic acid, threonine, creatine, guanine, serotonin, methionine, and leucine (when ranked on component 1; Supplementary Table S12). Correspondingly for **liver** samples, the most important metabolites included turanose, urea, thymine, lysine, myo-inositol, fructose-6-phosphate, sucrose, α-ketoglutaric acid, β-alanine, and cellobiose (Supplementary Table S13).

## Discussion

In this study we analysed the metabolic response of an amphibian host to chytridiomycosis with a focus on the small, polar metabolites amenable to analysis by gas chromatography. Our results clearly demonstrate marked effects of clinical chytridiomycosis on organism-wide metabolism, not limited to either the site of infection (skin) or effects on plasma electrolyte balance that had previously been described^10^. Clinical chytridiomycosis dramatically interrupted tissue homeostasis; affecting numerous biosynthetic and degradation pathways, and causing pronounced dysregulation of cellular energy metabolism in both the skin and liver tissues. We identified several key metabolites that may provide targets for research and intervention, and may help explain differences in susceptibility. The 20 key differentially abundant metabolites identified included α-ketoglutarate (2-oxoglutarate), isoleucine, pantothenate, serotonin, glutamate, myo-inositol, putrescine, 5HIAA, adenine, galactose, leucine, pyroglutamate, creatine, threonine, citrate, aspartate, tartrate, ribose, guanine, and adenosine (see Table 3 for key metabolite details). To our knowledge this is the first non-targeted metabolomics study of amphibian host skin and liver tissue responses to chytridiomycosis.

**Table 3:** Key metabolites in both skin and liver tissues discriminating between experimental groups (including sampling periods post exposure and populations), demonstrating overlap in significant results between univariate (ANOVA, t-test pattern matching and SAM) and multivariate analyses (PLS-DA, top 30 VIP scores ranked on component 1).

Group	Tissue	Metabolite identification (TMS groups, RT, RI)	ANOVA FDR	Tukey’s HSD	Pattern matching FDR	Correlation	SAM FDR	PLS-DA VIP comp. 1
Time-series	Skin	a-Ketoglutaric acid, x TMS, 23.95, 1578	1.05E-05	4-0; 4-1; 4-2; 4-3	0.005854	−0.50794	0	2.2876
		Serotonin, x TMS, 39.11, 2470	0.002533	4-0; 4-1; 4-2; 4-3			0	1.6147
		Putrescine, x TMS, 22.45, 1506_putative	0.036876	3-0; 4-0	0.005854	0.50557	0.00909	2.0726
		Adenine, 2 TMS, 29.74, 1869	0.036337	3-0; 4-0	0.005854	0.50493	0.00909	2.0262
		5-Hydroxyindole-3-acetic acid, 3 TMS, 35.49, 2212	0.002533	4-0; 4-1; 4-2; 4-3			0.000346	
		L-Threonine, 3 TMS, 19.59, 1387	0.02635	3-0; 4-0	0.012166	−0.472	0.003239	1.99
		L-Isoleucine, 2 TMS, 17.32, 1295	0.004158	4-0; 4-1; 4-2; 4-3			0.004607	
		DL-Tartaric acid 4TMS-like	0.021251	4-0; 4-1; 4-2	0.039136	−0.41978	0.00909	
		Glutamic acid, 3 TMS, 24.79, 1623	0.004834	4-0; 4-1; 4-2; 4-3	0.039136	−0.40884	0.003179	
		Adenosine, 4 TMS, 41.49, 2642			0.039136	0.41253	0.042185	
		Urea, 2 TMS, 16.14, 1249	0.02635	1-0; 3-1			0.000829	
		Serine, 2 TMS, 16.43, 1260	0.029108	3-0; 3-2			0.003179	1.719
		DL-Ornithine, 3 TMS, 24.71, 1623	0.012299	2-1; 3-2; 4-2			0.003293	
		L-Pyroglutamic acid, 2 TMS, 22.76, 1520_saturated	0.032369	4-0; 4-2	0.039136	−0.39293	0.019789	
		L-Lysine, 4 TMS, 30.54, 1915	0.036876	2-1; 3-1; 4-1			0.006945	1.7061
		Fumaric acid, 2 TMS, 18.29, 1357	0.02968	4-0; 4-3			0.027929	
		Aspartic acid, 2 TMS, 20.54, 1428					0.018362	1.651
		Cellobiose, x TMS, 42.19, 2962			0.040882	0.38103	0.039192	1.496
		g-Aminobutyric acid, 3 TMS, 22.9, 1526	0.0409	4-0; 4-1; 4-3			0.00909	
		Myo-Inositol, 6 TMS, 33.38, 2081			0.042804	−0.37252	0.032292	
	Liver	L-Isoleucine, 2 TMS, 17.32, 1295	3.51E-11	4-0; 2-1; 4-1; 3-2; 4-2; 4-3	4.15E-05	0.57373	0.003774	1.5512
		L-Glutamic acid, 2 TMS, 22.7, 1519*	2.86E-10	4-0; 4-1; 4-2; 4-3	2.50E-06	−0.6347	0.011043	1.5014
		5-Hydroxyindole-3-acetic acid, 3 TMS, 35.49, 2212	0.000582	4-0; 4-1; 4-2; 4-3	0.001556	0.45911	0.003029	2.5384
		D–Galactose, 5 TMS, MEOX, 29.94, 1880	0.025509	4-0; 4-1	0.011265	−0.38718	0.003029	2.6805
		L-Pyroglutamic acid, 2 TMS, 22.76, 1520_saturated	2.86E-10	4-0; 4-1; 4-2; 4-3	2.50E-06	−0.63459	0.011043	1.501
		Citric acid, 4 TMS, 28.69, 1817	9.67E-07	4-0; 4-1; 4-2; 4-3	0.000752	−0.4873	0.003029	1.966
		Putrescine, x TMS, 22.45, 1506_putative	5.64E-07	4-0; 4-1; 4-2; 4-3	6.71E-06	0.61232	0.003029	2.5008
		a-Ketoglutaric acid, x TMS, 23.95, 1578	8.29E-05	4-0; 4-1; 4-2; 4-3	0.002517	−0.44337	0.003029	1.9493
		L-Leucine, 2 TMS, 16.74, 1274	1.85E-08	4-0; 4-1; 3-2; 4-2; 4-3	4.18E-05	0.57127	0.011264	
		Creatine. x TMS, 23.39, 1551	6.32E-05	4-0; 4-1; 4-2; 4-3	0.000752	0.48784	0.003029	1.9563
		D-Fructose-6-phosphate 6TMS, MEOX, 36.72, 2300-put	5.97E-07	4-0; 4-1; 4-2; 4-3	0.002165	−0.44874	0.003029	1.8381
		g-Aminobutyric acid, 3 TMS, 22.9, 1526	4.26E-05	4-0; 4-1; 3-2; 4-2	0.000315	−0.51611	0.004805	1.7973
		D-Glucose-6-phosphate, 6 TMS, MEOX, 37.23, 2332	3.10E-06	4-0; 4-1; 4-2; 4-3	0.006038	−0.41173	0.003029	
		Fumaric acid, 2 TMS, 18.29, 1357	1.42E-06	4-0; 4-1; 4-2; 4-3	0.000934	−0.47769	0.005732	
		Serine, 3 TMS, 18.98, 1363	0.003442	3-0; 4-0; 4-1; 4-2	0.000661	0.49314	0.038474	
		L-Lysine, x TMS, 29.32, 1852	6.32E-05	4-0; 4-1; 4-2; 4-3	0.003008	0.4358	0.00402	1.5988
		Urea, 2 TMS, 16.14, 1249					0.011043	1.5188
		Glycine, 3 TMS, 17.63, 1308	0.048096	4-0	0.003008	0.43531		
		Sucrose, 8 TMS, 41.32, 2630.1	0.005833	1-0; 2-0; 3-1; 3-2			0.008361	
		Adenine, 2 TMS, 29.74, 1869	0.016867	4-0; 4-1; 4-2; 4-3			0.011043	
Site	Skin	Pantothenic acid, O,O,O-TMS-putative	0.012106	G-E; K-E	0.010965	−0.51381	0.002488	2.1179
		Myo-Inositol, 6 TMS, 33.38, 2081	0.017097	K-E; K-G			0.0199	
		L-Isoleucine, 2 TMS, 17.32, 1295	0.026777	K-E	0.010965	0.49005		
		L-Leucine, 2 TMS, 16.74, 1274	0.026777	K-E; K-G	0.010965	0.48099	0.039608	1.5988
		Creatine. x TMS, 23.39, 1551	0.046951	K-E; K-G	0.016922	0.45692	0.036566	1.8447
		Aspartic acid, 2 TMS, 20.54, 1428						1.9701
		L-Threonine, 2 TMS, 17.37, 1298						1.8778
		D–Ribose, 4 TMS, MEOX, 25.89, 1678			0.033096	0.42058		
		saturated_Guanine manual						1.7444
		Serotonin, x TMS, 39.11, 2470*						1.7352
	Liver	D–Turanose, 7 TMS, 42.29, 2702						2.7638
		Urea, 2 TMS, 16.14, 1249						2.507
		Thymine, 2 TMS, 19.94, 1403						2.4842
		L-Lysine, 3 TMS, 26.54, 1712						2.0644
		Myo-Inositol, 6 TMS, 33.38, 2081.1						1.8873
		D-Fructose-6-phosphate 6TMS, MEOX, 36.72, 2300-putative						1.7514
		Sucrose, 8 TMS, 41.32, 2630.1						1.734
		a-Ketoglutaric acid, x TMS, 23.95, 1578						1.6361
		beta-Alanine, 3TMS, 20.58, 1429						1.6332
		Cellobiose, x TMS, 42.19, 2962						1.4796

Comparisons found to be significant with post-hoc Tukey’s HSD tests are listed, as are the respective correlations for the pattern matching approach. Sham-exposed negative control group frogs were designated Group 0. Exposed/infected frogs sampled at various times post-exposure were designated Groups 1, 2, 3 and 4, corresponding to being sampled at 4, 8, 14 and 28–30 days post exposure, respectively. Source populations (sites) are as follows: Eucumbene, Grey Mare, Kiandra are represented by E, G and K respectively. Where metabolites were not found to be significantly different with a particular test, values have been omitted.

^*^Quantitation re-calculated from a lesser abundant ion (or isotope) within mass spectrum for accurate measurement of metabolites nearing the upper dynamic concentration of the MS.

The *L. v. alpina* metabolome of clinically diseased frogs was distinctly divergent from both unexposed control and subclinically Bd-infected frog groups, likely associated with end-stage pathophysiological changes occurring in the tissues shortly prior to disease-induced mortality^10^. The most important effect was the organism-wide disruption of cellular energy pathways in moribund frogs, particularly the tricarboxylic acid (TCA, Krebs or citric acid) cycle, glycolysis, and anaerobic fermentation pathways (Figs 3 and 4)^41^. The TCA cycle is central to cellular energy metabolism and also plays a role in providing substrates for numerous biosynthetic pathways. The function of the TCA cycle is to produce energy in the form of adenosine triphosphate (ATP) from the oxidation of acetyl-CoA^44^ which is primarily derived from dietary sources (sugars, fats and proteins) in the healthy fed state. Here we found that several key intermediates of the TCA cycle and their anaplerotic precursors were significantly depleted in skin and liver tissues from frogs of all populations showing clinical signs of chytridiomycosis (sampled at 28–30 days post-exposure), relative to negative control and subclinically infected frogs. These metabolites included α-ketoglutarate, glutamate, citrate, fumarate and aspartate (Fig. 1). In contrast, the remaining intermediates including pyruvate, acetyl CoA and succinyl CoA were not identified as significantly depleted and several of their anaplerotic precursors were found to be relatively elevated in both tissues (including isoleucine, leucine, glycine and lysine). Together, our findings suggest imbalance of TCA cataplerotic and anaplerotic processes, leading to disruption of energy production in the clinically diseased state^44^.

Within liver samples of clinically diseased frogs we also identified significant reduction in the relative concentration of three glucose precursors (galactose, fructose-6-phosphate and glucose-6-phosphate)^45^ compared with negative controls and subclinically infected frogs (Fig. 1B). As the liver is the key site for gluconeogenesis and a critical organ for energy metabolism^46^, these findings are consistent with organism-wide energy dysregulation in clinical chytridiomycosis. Anorexia (lack of appetite) is one of the first clinical signs of chytridiomycosis^47^. Together with infection-associated energy losses^48^, anorexia could lead to energy dysregulation in diseased frogs, and may contribute to the observed lethargy of the moribund state.

The accumulation of essential branched-chain amino acids (BCAAs) such as isoleucine and leucine in the moribund state is likely a by-product of tissue protein breakdown^49^ for gluconeogenesis or through toxic effects. Isoleucine was the most significantly elevated metabolite in the liver samples of clinically diseased frogs, but it was also significantly elevated in skin samples. Leucine was significantly elevated in the liver of clinically diseased frogs (Fig. 1). Unlike other amino acids, leucine and isoleucine have limited gluconeogenic capacity (ability to be converted to glucose for energy), and they are also not subject to hepatic metabolism^50^ as the liver lacks substantial branched chain amino-transferase enzyme for the conversion of BCAAs to branched chain α-ketoacids. For eventual elimination these BCAAs require terminal oxidation through the TCA cycle^44^, however, in dysregulated energy states as described above, this might be expected to lead to the resultant accumulation as determined here. Elevated BCAAs and the accumulation of their toxic intermediates is characteristic of organic acidurias (such as maple syrup urine disease), and these metabolic disorders have been linked with neurotoxicity and ketoacidosis among other signs^51^.

In contrast to elevated BCAAs, pathway analysis^41^ indicated that other amino acids were predominantly depleted in the moribund state and that their biosynthetic and degradation pathways were affected, particularly in skin tissues. In most cases this was linked with the depletion of α-ketoglutarate and glutamate. Additionally, the function of numerous other biosynthetic and degradation pathways was found to be compromised in clinically diseased animals, suggesting generalized failure of cellular homeostatic mechanisms and secondary functions. The most significantly affected pathways included nucleotide, carbohydrate, secondary metabolism, cofactor, and hormone biosynthesis and degradation as well as metabolic regulatory pathways (Fig. 3). Metabolites associated with detoxification pathways were also substantially depleted in the skin of moribund frogs, particularly those related to the synthesis of defense compounds with antibiotic effects, but also those associated with reactive oxygen species, acid resistance, and biological toxins. These findings are consistent with the production of antimicrobial peptides as demonstrated by other studies^12,52–54^, and they are also consistent with the failure to detoxify harmful products from the processes of inflammation and the pathogen itself (for example, several recent studies have characterized Bd metabolites with the capacity to inhibit lymphocyte responses^32,55,56^). Although studies have not demonstrated effects of chytridiomycosis on blood acid-base balance^57^, the evidence for reduced function of acid resistance pathways may be due to over utilization of buffering mechanisms due to excess BCAAs. Our results may indicate that systemic metabolic disruption due to infection with Bd may contribute to disease signs.

As an example of toxin accumulation, putrescine was found to be significantly elevated in the skin and liver of clinically diseased frogs in comparison with control and subclinically infected frogs (Fig. 1). Putrescine is a polyamine breakdown product of amino acids so named for its foul odour and association with decaying flesh^58^. As such, increased concentrations of putrescine may be an indicator of catabolism and were likely responsible for the putrid smell of infected individuals (as reported anecdotally by colleagues). Although putrescine could be associated with post-mortem autolysis during tissue collection, this cause is unlikely as putrescine was significantly positively correlated with sampling group (with controls from Group 0 exhibiting the lowest concentrations, regardless of actual sampling date). Putrescine is also active in immune processes and basic homeostatic mechanisms^59,60^. Interestingly, ornithine, and the polyamines putrescine and agmatine, are potent inducers of a trichothecine mycotoxin of *Fusarium graminearum*, whereby mycotoxin production is linked to the natural accumulation of these metabolites within the host^61^. These metabolites may thus also be tightly coupled to clinical signs of disease in moribund animals.

Common to several of the pathway disruptions we have discussed thus far is the severe depletion of both α-ketoglutarate and the amino acid glutamate. These metabolites were significantly depleted in both skin and liver tissues of clinically diseased frogs, with α-ketoglutarate identified as the metabolite with the most significantly decreased concentration in the skin samples, and glutamate the second most significantly reduced in the liver samples. Enzymatically linked pyroglutamate was also significantly depleted in both skin and liver tissues (Fig. 1). Alpha-ketoglutarate and glutamate are closely metabolically linked (bidirectional reaction catalyzed by glutamate dehydrogenase or transaminases), typically ubiquitous among tissues, and recent studies have confirmed α-ketoglutarate as a master regulatory metabolite^62–66^. Depletion of these metabolites may be both causal and a consequence of multiple pathophysiological features of clinical chytridiomycosis in the amphibian host, although it is as yet unknown whether depletion due to utilization by Bd may also be involved.

Glutamate itself functions as the most abundant excitatory neurotransmitter in the vertebrate nervous system^67^. Although we did not examine brain tissues, if glutamate depletion is systemic this could contribute to the loss of alertness and righting reflex observed in clinically diseased animals^68^. Alpha-ketoglutarate and glutamate are also precursors for GABA which was similarly found depleted in both skin and liver tissues (Fig. 1). GABA is an inhibitory neurotransmitter at neuronal synapses, and it can also be found in peripheral tissues unrelated to neurotransmission^69^. GABA has additionally been linked to pathogenicity and sporulation in some filamentous fungi^70^. In mammals, glutamate plays an important role in nitrogen elimination, acting as a substrate for the formation of urea via the urea cycle in the liver^64^. As a semi-aquatic amphibian, the alpine tree frog is likely to at least partly rely on direct ammonia excretion in place of urea formation for removing excess nitrogen, so the significance of interruption of the urea cycle here is unknown.

Glutamate is a precursor for nucleic acid and protein synthesis, including importantly collagen synthesis via the proline pathway^71^. Skin hypertrophy and hyperplasia has been an occasional histological finding in diseased frogs^72,73^, and utilization of glutamate for collagen synthesis in response to infection may be contributing to its depletion. Interestingly, although proline was one of our identified metabolites, we did not detect any significant differences in its accumulation during infection. Furthermore, glutamate is utilized during the synthesis of inflammatory acute phase proteins, which have been shown to accompany clinical chytridiomycosis^43^. Glutamate and α-ketoglutarate are also important for immune system function. Alpha-ketoglutarate has been called the ‘immune nutrient factor’ as it is a crucial energy precursor for rapidly dividing cells, particularly immune cells^66^. Glutamate has been demonstrated to promote phagocytosis, and both α-ketoglutarate and glutamate promote T helper 1 differentiation, favouring immune function via the production of interferon-γ, tumor-necrosis factor beta and interleukin-2^74^. We speculate that the dysregulation of metabolic pathways described above involving depleted α-ketoglutarate and glutamate may be driven primarily by an overwhelming but non-protective immune response in the later terminal stages of infection^43^.

We found several other metabolites that differed significantly between sampling groups. Serotonin was significantly reduced in the skin of clinically diseased frogs, while 5HIAA was elevated in both the skin and liver of diseased frogs (Fig. 1). Serotonin and 5HIAA are both intermediates of tryptophan metabolism and there is growing evidence that they play an important role in anti-fungal host defense mechanisms^75–78^. Serotonin occurs within the skin of many vertebrates, including frogs and fish^79^. In humans, serotonin is produced within cutaneous melanocytes, and through metabolic processes within the skin is acetylated and subsequently methylated to form N-acetylserotonin^80^ and melatonin, respectively. The significance of these findings concerning serotonin and 5HIAA is not yet clear but is currently the subject of further study.

Serine and threonine were both identified as reduced in the skin of clinically diseased frogs, while serine was elevated in the liver of clinically diseased frogs. Serine and threonine are small, closely related, polar, pH neutral, nucleophilic amino acids bearing a hydroxyl group. It is possible that part of the increase in these ubiquitous amino acids may have been associated with the fungal pathogen as serine proteases have been identified in Bd transcriptomics studies^81,82^.

We also compared metabolic responses between populations with differing long-term evolutionarily exposure histories and phenotypic susceptibility to chytridiomycosis. From the concurrent large survival experiment^42^, frogs from long-exposed population Kiandra were more resistant (survived significantly longer) when compared with frogs from the other populations. In this study we found that pantothenate, myo-inositol, isoleucine, leucine, and creatine demonstrated highly significantly different concentrations in skin samples between populations (Fig. 5). The pattern of strongest association identified (except for pantothenate and myo-inositol) was Eucumbene-Grey Mare-Kiandra, which unexpectedly suggests that the Bd-naive population Grey Mare lies between the other two in terms of metabolite accumulation, in contrast to the clinical survival results. Univariate analyses revealed no metabolites with significantly different concentrations between populations in the liver samples. This was likely due to the liver’s central role as an important homeostatic organ in all populations, and the skin being more susceptible to external evolutionary selection pressures than the other organs due to fungal penetration.

Pantothenate demonstrated the most significant difference (across all analyses) in relative concentration between source populations, with skin samples from Eucumbene frogs having higher concentrations than samples from both Grey Mare and Kiandra. Pantothenate (otherwise known as vitamin B_5_) is an essential water-soluble vitamin for the synthesis of coenzyme-A (CoA) which is principally involved in energy (tricarboxylic acid cycle) and fatty acid metabolism. This metabolite is also essential for the growth of pathogenic organisms, including fungi^83^, and has been previously measured in developing frogs^84^. Analogues of pantothenate have even shown antimicrobial capabilities, as they prevent the uptake of the naturally occurring endogenous and essential metabolites^85,86^. Whilst there is no determined time series association, different amounts within the skin among frog populations may imply a selective pressure towards this difference. It is unclear, however, how differences in pantothenic acid relate to the clinical evidence of survival between the populations, since in the clutches we compared, frogs from Eucumbene were more susceptible to infection than those from Kiandra.

Interestingly, the metabolite composition of the skin (site of Bd infection) and liver tissues differed substantially, consistent with the underlying differential physiology and biochemistry of these tissues, regardless of health or disease status (Supplementary Fig. S1). The skin tissue uniquely contained guanine, while the liver tissue was considerably richer in sugars and sugar phosphate metabolites. Guanine was identified as elevated in skin samples from Eucumbene and decreased in samples from Kiandra. This metabolite has been long known for its role as a pigment in the skin of amphibians^87,88^ and more recently characterized for its light reflective properties in the scales of fish^89^ and chameleons^90^. As a point of interest, the other primary amphibian iridophore pigments^87^ (purines hypoxanthine and adenine) were also identified in the *L. v. alpina* skin.

Many metabolites are ubiquitous in life, particularly intermediates of primary metabolism. Whilst the presence or absence of specific metabolites can be inferred from biochemical pathways annotated from the genome, transcriptome or proteome, the metabolome is tightly coupled to the environment and resulting phenotype. Metabolites observed within diseased tissues may originate from the host or pathogen, as well as commensal organisms in superficial (mucosae or epidermis) or gastrointestinal tissues. Metabolites of non-host origin could not all be unambiguously distinguished from the endogenous host metabolites with the methods utilized here yet this distinction in future pathogenesis studies may be worthwhile (for example, to evaluate the release of toxic fungal or bacterial metabolites). Here, we sought to clarify the origin by comparing the metabolite profiles of the host to that of Bd in culture (data not shown) for an exclusionary approach to dissecting the origin of metabolites as frog or fungal, although the expression of the fungal metabolome will likely differ between an *in vitro* and parasitic life cycle. Unsurprisingly a select few metabolites were specific to only one or other of the frog tissues, including the measured sugar phosphates, which were associated with liver tissues, and other metabolites with roles outside of primary metabolism. Although, the influence of fungal metabolites within the measurements was likely negligible compared to that of the frog tissues, these measurements are nonetheless important for dissecting this pathosystem.

Depending on the regulatory mechanisms, and proximate metabolite source or relationship to gene and protein expression, a systems biology approach may greatly assist in understanding these complex host-pathogen relationships. Future approaches could include (1) comparing the metabolomes of infected frogs, with those that have been treated with an antibiotic to reduce or eliminate bacterial load, (2) biolabeling (for example, using visual or otherwise detectable tags, such as green fluorescent protein or stable isotopic labelling), or alternatively (3) mass spectrometry imaging to examine the source of metabolites. The concentration of metabolites in tissues is also reliant on their flux; the rate of production (upstream processes), but also their rate of modification, anabolism or catabolism to other metabolites, or removal from the system^91^. The inclusion of fluxomics data would also likely provide further detail of metabolite origin. Further work on assessing the importance of the specific metabolites (particularly α-ketoglutarate, glutamate, serotonin, putrescine, pantothenate, serine and threonine) could include examination of the tissue metabolome of other amphibian species. Further studies could also include targeted functional validation studies to characterize metabolite role by blocking production within the host, inhibiting activity within skin explants *ex vivo*, or testing the effect on Bd growth *in vitro*^92^.

To our knowledge, this study was the first to apply a non-targeted metabolomics approach to a fungal wildlife disease and specifically to dissect the host-pathogen interface through analysis of host tissues. We demonstrated that the metabolome of clinically diseased frogs diverged distinctly from that of subclinically Bd-infected frogs and unexposed control groups. We found that chytridiomycosis dramatically affected organism-wide homeostatic mechanisms, including interruption of biosynthetic and degradation pathways as well as causing dysregulation of cellular energy metabolism. Depletion of α-ketoglutarate and glutamate appeared central to these changes. This was also uniquely the first metabolomics investigation of the alpine tree frog, and as such we have revealed numerous interesting observations on the composition of the *L. v. alpina* skin and liver tissues. Metabolites relating to differences in the population of origin, which may be associated with variation in phenotypic resistance or tolerance between populations were also uncovered. Most notably, the differences in metabolite expression that we have identified may be characteristic of processes involved in chytridiomycosis across host species, and may have broad relevance to understanding the pathogenesis of fungal skin diseases and future control of devastating fungal pathogens such as Bd.

## Electronic supplementary material

Supplementary Information
