# Supplementary material for: Chytridiomycosis causes catastrophic organism-wide metabolic dysregulation including profound failure of cellular energy pathways
Source: Sci Rep. 2018 May 29;8:8188. doi: 10.1038/s41598-018-26427-z (PMC5974026; doi:10.1038/s41598-018-26427-z)
Supplement: Supplementary file 1 — Supplementary Information [file 41598_2018_26427_MOESM1_ESM.docx]

**SUPPLEMENTARY INFORMATION**

**“Chytridiomycosis causes catastrophic organism-wide metabolic dysregulation including profound failure of cellular energy pathways”**

Laura F. Grogan, Lee F. Skerratt, Lee Berger, Scott D. Cashins,

Robert D. Trengove & Joel P. A. Gummer

### CONTENTS

[CONTENTS 2](#_Toc503766626)

[SUPPLEMENTARY FIGURES 3](#_Toc503766627)

[Figure S1. Principal components analysis scores plot for metabolite expression from both liver and skin tissue samples. 3](#_Toc503766628)

[SUPPLEMENTARY TABLES 4](#_Toc503766629)

[Table S1. One-way ANOVA Tukey's honestly significant difference post-hoc tests on skin samples to determine significant comparisons between sampling periods 4](#_Toc503766630)

[Table S2. Pattern searching template matching approach (Groups 0-4 in order) on skin samples to determine metabolites with significant comparisons between sampling periods (only showing metabolites with FDR < 0.05). 4](#_Toc503766631)

[Table S3. Significance Analysis of Microarrays on skin samples to determine metabolites with significant differences between sampling periods (only showing metabolites with FDR < 0.05). 5](#_Toc503766632)

[Table S4. One-way ANOVA Tukey's honestly significant difference post-hoc tests on liver samples to determine significant comparisons between sampling periods 6](#_Toc503766633)

[Table S5. Pattern searching template matching approach (Groups 0-4 in order) on liver samples to determine metabolites with significant comparisons between sampling periods (only showing metabolites with FDR < 0.05). 7](#_Toc503766634)

[Table S6. Significance Analysis of Microarrays on liver samples to determine metabolites with significant differences between sampling periods (only showing metabolites with FDR < 0.05). 8](#_Toc503766635)

[Table S7. Variable Importance in Projection measures of the top 30 ranked metabolites (sorted by component one) from Partial Least Squares discriminant analysis (PLS-DA) of skin samples, grouping by sample period. 9](#_Toc503766636)

[Table S8. Variable Importance in Projection measures of the top 30 ranked metabolites (sorted by component one) from Partial Least Squares discriminant analysis (PLS-DA) of liver samples, grouping by sample period. 10](#_Toc503766637)

[Table S9. One-way ANOVA Tukey's honestly significant difference post-hoc tests on skin samples to determine significant comparisons between populations (only showing metabolites with FDR < 0.05). 10](#_Toc503766638)

[Table S10. Pattern searching template matching approach (Eucumbene-Grey Mare-Kiandra) on skin samples to determine analytes with significant comparisons between populations (only showing metabolites with FDR < 0.05). 10](#_Toc503766639)

[Table S11. Significance Analysis of Microarrays on skin samples to determine metabolites with significant differences between populations (only showing metabolites with FDR < 0.05). 11](#_Toc503766640)

[Table S12. Variable Importance in Projection measures of the top 30 ranked metabolites (sorted by component one) from Partial Least Squares discriminant analysis (PLS-DA) of skin samples, grouping by population. 11](#_Toc503766641)

[Table S13. Variable Importance in Projection measures of the top 30 ranked metabolites (sorted by component one) from Partial Least Squares discriminant analysis (PLS-DA) of liver samples, grouping by population. 11](#_Toc503766642)

[REFERENCES 12](#_Toc503766643)

### SUPPLEMENTARY FIGURES

Figure S1. Principal components analysis scores plot for metabolite expression from both liver and skin tissue samples. Expression data was first normalized by Total Area under Chromatogram by sample, and overall dataset log transformation and pareto scaling. Samples separate by tissue type on the horizontal axis (Principal Component 1, representing 31% of the variability) with liver samples on the left (‘Li’ in sample name) and skin samples on the right (‘Sk’ in sample name). Samples separate only mildly by batch (date of processing) on the vertical axis (Principal Component 2, representing 10.2% of the variability), consistent with preventative GC-MS maintenance regimens. Reprinted with permission from Grogan et al. 2018.

**
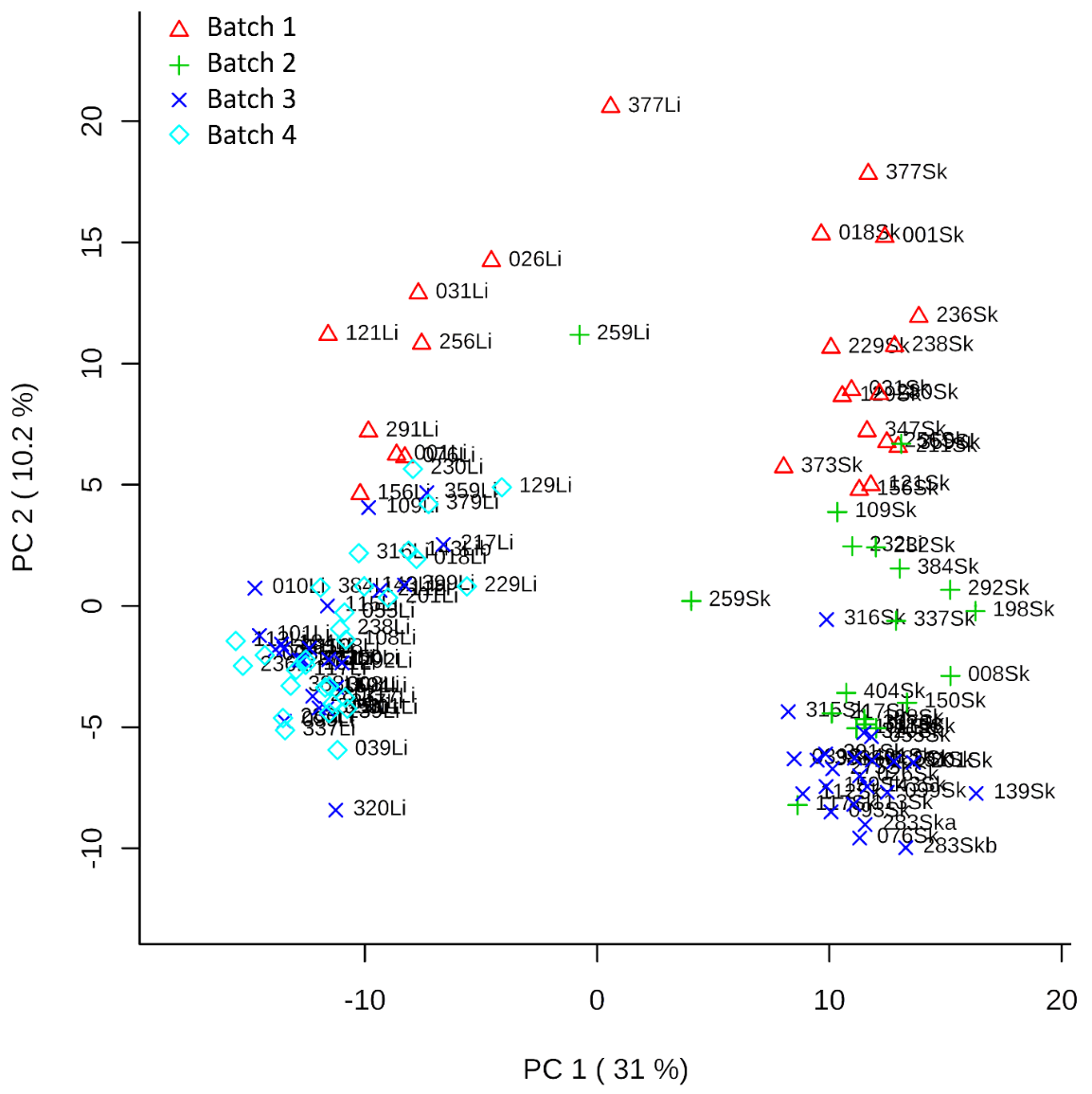
**

### SUPPLEMENTARY TABLES

Table S1. One-way ANOVA Tukey's honestly significant difference post-hoc tests on skin samples to determine significant comparisons between sampling periods (only showing metabolites with FDR < 0.05). The last column (Tukey's HSD) represents the sample Group comparisons that yielded significant results. Sham-exposed negative control group frogs were designated Group 0. Exposed/infected frogs sampled at various times post-exposure were designated Groups 1, 2, 3 and 4, corresponding to being sampled at 4, 8, 14 and 28-30 days post exposure, respectively. Metabolites with differences between sampling groups excluding the clinically diseased group (4) are highlighted in blue.

| # | Metabolite name | f.value | p.value | -log10(p) | FDR | Tukey's HSD |
| --- | --- | --- | --- | --- | --- | --- |
| 1 | a-Ketoglutaric acid, x TMS, 23.95, 1578 | 14.98 | 5.15E-08 | 7.2884 | 1.05E-05 | 4-0; 4-1; 4-2; 4-3 |
| 2 | Unknown_21.778, 1476, m/z234_arabino-hexos-2-ulose-like | 9.1607 | 1.39E-05 | 4.8558 | 0.001136 | 4-0; 4-1; 4-2; 4-3 |
| 3 | Mix J Unknown 3, 21.95, 1484_malic acid-like | 8.9886 | 1.68E-05 | 4.775 | 0.001136 | 4-0; 4-1; 4-2; 4-3 |
| 4 | Mix C Unknown 3, 24.71, 1617 | 7.8045 | 6.27E-05 | 4.2025 | 0.002533 | 4-0; 4-1; 4-2; 4-3 |
| 5 | Serotonin, x TMS, 39.11, 2470 | 7.769 | 6.53E-05 | 4.1849 | 0.002533 | 4-0; 4-1; 4-2; 4-3 |
| 6 | 5-Hydroxyindole-3-acetic acid, 3 TMS, 35.49, 2212 | 7.65 | 7.49E-05 | 4.1257 | 0.002533 | 4-0; 4-1; 4-2; 4-3 |
| 7 | Unknown_23.546, 1558, m/z 292-threonic acid-like | 7.0164 | 0.000157 | 3.8051 | 0.004158 | 4-0; 4-1; 4-2; 4-3 |
| 8 | L-Isoleucine, 2 TMS, 17.32, 1295 | 6.9784 | 0.000164 | 3.7855 | 0.004158 | 4-0; 4-1; 4-2; 4-3 |
| 9 | Glutamic acid, 3 TMS, 24.79, 1623 | 6.7341 | 0.000219 | 3.6593 | 0.004834 | 4-0; 4-1; 4-2; 4-3 |
| 10 | Serotonin, x TMS, 39.11, 2470* | 6.6646 | 0.000238 | 3.6232 | 0.004834 | 4-0; 4-1; 4-2; 4-3 |
| 11 | Glutamic acid, 3 TMS, 24.79, 1623* | 6.5332 | 0.000279 | 3.5546 | 0.004909 | 4-0; 4-1; 4-2; 4-3 |
| 12 | Glutamic acid, 3 TMS, 24.79, 1623* | 6.5004 | 0.00029 | 3.5374 | 0.004909 | 4-0; 4-1; 4-2; 4-3 |
| 13 | DL-Ornithine, 3 TMS, 24.71, 1623 | 5.6882 | 0.000788 | 3.1037 | 0.012299 | 2-1; 3-2; 4-2 |
| 14 | Unknown_39.602, 2501, m/z174-serotonin-like | 5.5097 | 0.000986 | 3.0063 | 0.014293 | 4-0; 4-1; 4-2; 4-3 |
| 15 | DL-Tartaric acid 4TMS-like | 5.1432 | 0.00157 | 2.804 | 0.021251 | 4-0; 4-1; 4-2 |
| 16 | Urea, 2 TMS, 16.14, 1249 | 4.9015 | 0.002143 | 2.669 | 0.02635 | 1-0; 3-1 |
| 17 | L-Threonine, 3 TMS, 19.59, 1387 | 4.8789 | 0.002207 | 2.6563 | 0.02635 | 3-0; 4-0 |
| 18 | Serine, 2 TMS, 16.43, 1260 | 4.7582 | 0.002581 | 2.5882 | 0.029108 | 3-0; 3-2 |
| 19 | L-Glutamic acid, 2 TMS, 22.7, 1519* | 4.7048 | 0.002767 | 2.558 | 0.029561 | 4-0; 4-2 |
| 20 | Fumaric acid, 2 TMS, 18.29, 1357 | 4.6624 | 0.002924 | 2.534 | 0.02968 | 4-0; 4-3 |
| 21 | Mix N Unknown 1, 22.98, 1532 | 4.5518 | 0.00338 | 2.4711 | 0.031601 | 4-0; 4-2 |
| 22 | L-Glutamic acid, 2 TMS, 22.7, 1519* | 4.5188 | 0.00353 | 2.4523 | 0.031601 | 4-0; 4-2 |
| 23 | L-Threonine, 2 TMS, 17.37, 1298 | 4.5079 | 0.003581 | 2.4461 | 0.031601 | 3-0; 3-2 |
| 24 | Unknown, x TMS, 22.73, 1520* | 4.4449 | 0.00389 | 2.4101 | 0.032369 | 4-0; 4-2 |
| 25 | L-Pyroglutamic acid, 2 TMS, 22.76, 1520* | 4.3867 | 0.0042 | 2.3768 | 0.032369 | 4-0; 4-2 |
| 26 | L-Glutamic Acid, 2 TMS, 22.75, 1519* | 4.3693 | 0.004298 | 2.3668 | 0.032369 | 4-0; 4-2 |
| 27 | L-Pyroglutamic acid, 2 TMS, 22.76, 1520* | 4.3679 | 0.004305 | 2.366 | 0.032369 | 4-0; 4-2 |
| 28 | Mix F Unknown 1, 19.28, 1377 | 4.3077 | 0.004662 | 2.3314 | 0.033802 | 4-0; 4-1; 4-2 |
| 29 | Adenine, 2 TMS, 29.74, 1869 | 4.2267 | 0.005191 | 2.2848 | 0.036337 | 3-0; 4-0 |
| 30 | Putrescine, x TMS, 22.45, 1506* | 4.1693 | 0.005603 | 2.2516 | 0.036876 | 3-0; 4-0 |
| 31 | L-Lysine, 4 TMS, 30.54, 1915 | 4.1655 | 0.005631 | 2.2494 | 0.036876 | 2-1; 3-1; 4-1 |
| 32 | g-Aminobutyric acid, 3 TMS, 22.9, 1526 | 4.064 | 0.006447 | 2.1906 | 0.0409 | 4-0; 4-1; 4-3 |
| 33 | Uridine, 3 TMS, 38.98, 2462 | 3.9073 | 0.007954 | 2.0994 | 0.04893 | 4-0; 4-1; 4-2 |
|  |  |  |  |  |  |  |

*Additional quantitation calculated from lesser abundant ion, to account for MS detector saturation. If ‘unknown’ metabolite, a metabolite with the closest fragmentation match is provided in brackets.

#### Table S2. Pattern searching template matching approach (Groups 0-4 in order) on skin samples to determine metabolites with significant comparisons between sampling periods (only showing metabolites with FDR < 0.05).

| # | Metabolite name | correlation | t-stat | p-value | FDR |
| --- | --- | --- | --- | --- | --- |
| 1 | Unknown_21.778, 1476, m/z234_arabino-hexos-2-ulose-like | -0.51429 | -4.2826 | 8.17E-05 | 0.005854 |
| 2 | a-Ketoglutaric acid, x TMS, 23.95, 1578 | -0.50794 | -4.2111 | 0.000103 | 0.005854 |
| 3 | Putrescine, x TMS, 22.45, 1506* | 0.50557 | 4.1847 | 0.000113 | 0.005854 |
| 4 | Adenine, 2 TMS, 29.74, 1869 | 0.50493 | 4.1776 | 0.000115 | 0.005854 |
| 5 | Unknown_23.546, 1558, m/z 292-threonic acid-like | -0.49706 | -4.0909 | 0.000153 | 0.006212 |
| 6 | L-Threonine, 3 TMS, 19.59, 1387 | -0.472 | -3.8235 | 0.00036 | 0.012166 |
| 7 | Mix C Unknown 3, 24.71, 1617 | -0.46039 | -3.7037 | 0.000523 | 0.015165 |
| 8 | DL-Tartaric acid 4TMS-like | -0.41978 | -3.303 | 0.001753 | 0.039136 |
| 9 | Adenosine, 4 TMS, 41.49, 2642 | 0.41253 | 3.2341 | 0.002143 | 0.039136 |
| 10 | L-Glutamic acid, 2 TMS, 22.7, 1519* | -0.40884 | -3.1993 | 0.00237 | 0.039136 |
| 11 | Mix F Unknown 1, 19.28, 1377 | -0.40386 | -3.1527 | 0.002709 | 0.039136 |
| 12 | L-Glutamic acid, 2 TMS, 22.7, 1519* | -0.40033 | -3.1198 | 0.002976 | 0.039136 |
| 13 | Unknown, x TMS, 22.73, 1520* | -0.39673 | -3.0865 | 0.00327 | 0.039136 |
| 14 | L-Glutamic Acid, 2 TMS, 22.75, 1519* | -0.3935 | -3.0567 | 0.003557 | 0.039136 |
| 15 | L-Pyroglutamic acid, 2 TMS, 22.76, 1520* | -0.39293 | -3.0516 | 0.003609 | 0.039136 |
| 16 | L-Pyroglutamic acid, 2 TMS, 22.76, 1520* | -0.39291 | -3.0513 | 0.003611 | 0.039136 |
| 17 | Mix N Unknown 1, 22.98, 1532 | -0.39265 | -3.0489 | 0.003636 | 0.039136 |
| 18 | Putrescine, 4 TMS, 27.09, 1739* | 0.38681 | 2.9955 | 0.004221 | 0.039136 |
| 19 | Putrescine, 4 TMS, 27.09, 1739* | 0.38663 | 2.9939 | 0.004241 | 0.039136 |
| 20 | Putrescine, 4 TMS, 27.09, 1739 | 0.38642 | 2.992 | 0.004262 | 0.039136 |
| 21 | Putrescine, 4 TMS, 27.09, 1739* | 0.38642 | 2.992 | 0.004262 | 0.039136 |
| 22 | Putrescine, 4 TMS, 27.13, 1738* | 0.38642 | 2.992 | 0.004262 | 0.039136 |
| 23 | L-Threonine, 2 TMS, 17.37, 1298 | -0.38486 | -2.9778 | 0.004434 | 0.039136 |
| 24 | Cellobiose, x TMS, 42.19, 2962 | 0.38103 | 2.9431 | 0.00488 | 0.040882 |
| 25 | Glutamic acid, 3 TMS, 24.79, 1623 | -0.37978 | -2.9318 | 0.005035 | 0.040882 |
| 26 | likely artifact-glucose-like | -0.37436 | -2.8831 | 0.005752 | 0.042804 |
| 27 | likely artifact-glucose-like.1 | -0.37436 | -2.8831 | 0.005752 | 0.042804 |
| 28 | Myo-Inositol, 6 TMS, 33.38, 2081 | -0.37252 | -2.8667 | 0.006015 | 0.042804 |
| 29 | Glutamic acid, 3 TMS, 24.79, 1623* | -0.37023 | -2.8462 | 0.006358 | 0.042804 |
| 30 | Glutamic acid, 3 TMS, 24.79, 1623* | -0.3697 | -2.8415 | 0.006439 | 0.042804 |
| 31 | Unknown_46.866, 3064, m/z169 | 0.36907 | 2.8359 | 0.006537 | 0.042804 |
|  |  |  |  |  |  |

*Additional quantitation calculated from lesser abundant ion, to account for MS detector saturation

#### Table S3. Significance Analysis of Microarrays on skin samples to determine metabolites with significant differences between sampling periods (only showing metabolites with FDR < 0.05).

| # | Metabolite name | d.value | stdev | rawp | FDR |
| --- | --- | --- | --- | --- | --- |
| 1 | a-Ketoglutaric acid, x TMS, 23.95, 1578 | 8.9331 | 0.82688 | 0 | 0 |
| 2 | Serotonin, x TMS, 39.11, 2470 | 5.9359 | 1.8124 | 0 | 0 |
| 3 | Unknown_21.778, 1476, m/z234_arabino-hexos-2-ulose | 5.8675 | 0.9972 | 0 | 0 |
| 4 | Mix C Unknown 3, 24.71, 1617 | 5.3872 | 1.2473 | 0 | 0 |
| 5 | 5-Hydroxyindole-3-acetic acid, 3 TMS, 35.49, 2212 | 5.2971 | 1.26 | 4.93E-05 | 0.000346 |
| 6 | Serotonin, x TMS, 39.11, 2470* | 5.2228 | 2.0274 | 4.93E-05 | 0.000346 |
| 7 | Unknown_39.602, 2501, m/z174-serotonin-like | 4.4489 | 2.3475 | 0.000197 | 0.000829 |
| 8 | Unknown_23.546, 1558, m/z 292-threonic acid-like | 4.4213 | 0.95352 | 0.000197 | 0.000829 |
| 9 | Mix J Unknown 3, 21.95, 1484_malic acid-like | 4.3858 | 0.53329 | 0.000197 | 0.000829 |
| 10 | Urea, 2 TMS, 16.14, 1249 | 4.2497 | 3.6491 | 0.000197 | 0.000829 |
| 11 | Glutamic acid, 3 TMS, 24.79, 1623 | 3.5756 | 0.63361 | 0.001035 | 0.003179 |
| 12 | Serine, 2 TMS, 16.43, 1260 | 3.5387 | 1.6241 | 0.001035 | 0.003179 |
| 13 | Glutamic acid, 3 TMS, 24.79, 1623* | 3.5069 | 0.64856 | 0.001084 | 0.003179 |
| 14 | Glutamic acid, 3 TMS, 24.79, 1623* | 3.4953 | 0.65097 | 0.001133 | 0.003179 |
| 15 | Mix N Unknown 1, 22.98, 1532 | 3.4929 | 1.8461 | 0.001133 | 0.003179 |
| 16 | L-Threonine, 2 TMS, 17.37, 1298 | 3.4301 | 1.7814 | 0.001232 | 0.003239 |
| 17 | DL-Ornithine, 3 TMS, 24.71, 1623 | 3.4033 | 0.83362 | 0.00133 | 0.003293 |
| 18 | L-Isoleucine, 2 TMS, 17.32, 1295 | 3.2503 | 0.48796 | 0.00197 | 0.004607 |
| 19 | L-Lysine, 4 TMS, 30.54, 1915 | 3.0761 | 1.5805 | 0.003153 | 0.006945 |
| 20 | L-Threonine, 3 TMS, 19.59, 1387 | 3.0353 | 0.92147 | 0.003301 | 0.006945 |
| 21 | DL-Tartaric acid 4TMS-like | 2.8166 | 0.67758 | 0.005123 | 0.00909 |
| 22 | Putrescine, x TMS, 22.45, 1506_putative | 2.7989 | 1.1431 | 0.005222 | 0.00909 |
| 23 | Adenine, 2 TMS, 29.74, 1869 | 2.794 | 1.0915 | 0.00532 | 0.00909 |
| 24 | g-Aminobutyric acid, 3 TMS, 22.9, 1526 | 2.7928 | 1.2296 | 0.00532 | 0.00909 |
| 25 | Unknown_24.994, 1631, m/z174.1 | 2.772 | 1.6201 | 0.005616 | 0.00909 |
| 26 | Unknown_24.994, 1631, m/z174 | 2.772 | 1.6201 | 0.005616 | 0.00909 |
| 27 | Mix F Unknown 1, 19.28, 1377 | 2.7386 | 0.97687 | 0.005961 | 0.009291 |
| 28 | Unknown_46.866, 3064, m/z169 | 2.7296 | 3.3207 | 0.006207 | 0.009329 |
| 29 | Unknown_46.848, 3063, m/z169 | 2.65 | 3.3413 | 0.007537 | 0.010938 |
| 30 | Unknown_26.066, 1685, m/z159 | 2.4952 | 2.2908 | 0.010739 | 0.015065 |
| 31 | Unknown_35.898, 2236, m/z188-putative phosphoric a | 2.4411 | 1.3084 | 0.011773 | 0.015983 |
| 32 | Unknown_22.954, 1529, m/z 160 | 2.3848 | 2.38 | 0.013054 | 0.017168 |
| 33 | Unknown_38.398, 2421, m/z387-myo-inositol-like | 2.3334 | 1.4162 | 0.014532 | 0.018362 |
| 34 | Aspartic acid, 2 TMS, 20.54, 1428 | 2.3212 | 2.1466 | 0.015074 | 0.018362 |
| 35 | L-Glutamic acid, 2 TMS, 22.7, 1519* | 2.3093 | 0.53953 | 0.015271 | 0.018362 |
| 36 | L-Glutamic acid, 2 TMS, 22.7, 1519* | 2.2339 | 0.54718 | 0.017241 | 0.019789 |
| 37 | Unknown, x TMS, 22.73, 1520* | 2.2049 | 0.55095 | 0.018374 | 0.019789 |
| 38 | L-Pyroglutamic acid, 2 TMS, 22.76, 1520* | 2.1888 | 0.55738 | 0.018916 | 0.019789 |
| 39 | L-Pyroglutamic acid, 2 TMS, 22.76, 1520* | 2.1764 | 0.55581 | 0.019754 | 0.019789 |
| 40 | L-Glutamic Acid, 2 TMS, 22.75, 1519* | 2.1759 | 0.55523 | 0.019754 | 0.019789 |
| 41 | Uridine, 3 TMS, 38.98, 2462 | 2.1756 | 0.70317 | 0.019754 | 0.019789 |
| 42 | D--Galactose, 5 TMS, MEOX, 29.94, 1880 | 2.1616 | 1.6206 | 0.020148 | 0.019789 |
| 43 | DL-Ornithine, 4 TMS, 28.65, 1815 | 2.1458 | 0.78311 | 0.02064 | 0.019789 |
| 44 | Unknown_39.274, 2478, m/z204 | 2.1452 | 1.6529 | 0.02069 | 0.019789 |
| 45 | Unknown_41.866, 2664, m/z204 | 2.0404 | 1.2098 | 0.026207 | 0.024509 |
| 46 | Unknown_37.130, 2142, m/z315-myo-inositol-2-phosph | 2.0077 | 1.2899 | 0.027882 | 0.025509 |
| 47 | Glycine, 2 TMS, 12.72, 1110 | 1.9923 | 2.0406 | 0.028966 | 0.025936 |
| 48 | Putrescine, 4 TMS, 27.09, 1739* | 1.915 | 1.5238 | 0.034581 | 0.027929 |
| 49 | Putrescine, 4 TMS, 27.09, 1739* | 1.9115 | 1.5234 | 0.034926 | 0.027929 |
| 50 | Putrescine, 4 TMS, 27.13, 1738* | 1.9094 | 1.5233 | 0.035025 | 0.027929 |
| 51 | Putrescine, 4 TMS, 27.09, 1739* | 1.9094 | 1.5233 | 0.035025 | 0.027929 |
| 52 | Putrescine, 4 TMS, 27.09, 1739 | 1.9094 | 1.5233 | 0.035025 | 0.027929 |
| 53 | Fumaric acid, 2 TMS, 18.29, 1357 | 1.9049 | 0.38663 | 0.035172 | 0.027929 |
| 54 | Unknown_20.426, 1421, m/z 172 | 1.8655 | 1.0017 | 0.037389 | 0.029139 |
| 55 | Unknown_36.722, 2295, m/z318-myo-inositol-like | 1.8061 | 1.3493 | 0.041823 | 0.032002 |
| 56 | Unknown_29.466, 1855, m/z217 | 1.7976 | 0.8643 | 0.042956 | 0.032006 |
| 57 | Pantothenic acid, O,O,O-TMS-putative | 1.7937 | 1.1638 | 0.04335 | 0.032006 |
| 58 | Unknown, m/z_320 | 1.765 | 3.7282 | 0.045172 | 0.032292 |
| 59 | Myo-Inositol, 6 TMS, 33.38, 2081 | 1.7641 | 0.75554 | 0.045271 | 0.032292 |
| 60 | D-Fructose-6-phosphate 6TMS, MEOX, 36.72, 2300-put | 1.7387 | 1.6023 | 0.04798 | 0.033654 |
| 61 | Ribose-5-phosphate, 5 TMS, MEOX, 33.65, 2100 | 1.7101 | 0.92538 | 0.051182 | 0.035311 |
| 62 | Unknown_42.354, 2698, m/z289.1 | 1.6737 | 1.6426 | 0.054926 | 0.037283 |
| 63 | Cellobiose, x TMS, 42.19, 2962 | 1.6432 | 1.1754 | 0.05867 | 0.039192 |
| 64 | Adenosine, 4 TMS, 41.49, 2642 | 1.5811 | 0.75195 | 0.065714 | 0.042185 |
| 65 | L-Lysine, x TMS, 29.32, 1852 | 1.5809 | 0.97429 | 0.065714 | 0.042185 |
| 66 | Unknown_34.202, 2129, m/z352-putative guanine | 1.5797 | 5.9778 | 0.066158 | 0.042185 |
| 67 | Unknown_18.090, 1327, m/z 315 | 1.568 | 0.46915 | 0.067783 | 0.042577 |
| 68 | Unknown_31.242, 1953, m/z361-cellobiose-like | 1.5022 | 1.0864 | 0.077882 | 0.048201 |
| 69 | D-Fructose-1-phosphate, x TMS, 36.58, 2290 | 1.4867 | 1.3363 | 0.080739 | 0.049109 |
| 70 | L-Methionine, 1 TMS, 20.27, 1416_putative.2 | 1.4621 | 2.37 | 0.084828 | 0.049109 |
| 71 | Unknown_30.49, 1908, m/z 174-dopamine-like | 1.4585 | 1.1398 | 0.085468 | 0.049109 |
| 72 | Unknown_53.63, 3702, m/z311-stearic acid-like | 1.4574 | 1.9411 | 0.085764 | 0.049109 |
| 73 | likely artifact-glucose-like.1 | 1.4451 | 1.2034 | 0.088227 | 0.049109 |
| 74 | likely artifact-glucose-like | 1.4451 | 1.2034 | 0.088227 | 0.049109 |
| 75 | L-Tyrosine, 3 TMS, 30.86, 1934 | 1.442 | 1.3308 | 0.088916 | 0.049109 |
| 76 | Citric acid, 4 TMS, 28.69, 1817 | 1.4419 | 1.1849 | 0.088916 | 0.049109 |
| 77 | Unknown_42.354, 2704, m/z217 | 1.4374 | 1.6519 | 0.089852 | 0.049109 |
|  |  |  |  |  |  |

*Additional quantitation calculated from lesser abundant ion, to account for MS detector saturation

Table S4. One-way ANOVA Tukey's honestly significant difference post-hoc tests on liver samples to determine significant comparisons between sampling periods (only showing metabolites with FDR < 0.05). The last column (Tukey's HSD) represents the sample Group comparisons that yielded significant results. Sham-exposed negative control group frogs were designated Group 0. Exposed/infected frogs sampled at various times post-exposure were designated Groups 1, 2, 3 and 4, corresponding to being sampled at 4, 8, 14 and 28-30 days post exposure, respectively. Metabolites with differences between sampling groups excluding the clinically diseased group (4) are highlighted in blue.

| # | Metabolite name | f.value | p.value | -log10(p) | FDR | Tukey's HSD |
| --- | --- | --- | --- | --- | --- | --- |
| 1 | L-Isoleucine, 2 TMS, 17.32, 1295 | 30.871 | 1.75E-13 | 12.757 | 3.51E-11 | 4-0; 2-1; 4-1; 3-2; 4-2; 4-3 |
| 2 | L-Glutamic acid, 2 TMS, 22.7, 1519* | 25.259 | 6.61E-12 | 11.179 | 2.86E-10 | 4-0; 4-1; 4-2; 4-3 |
| 3 | Unknown, x TMS, 22.73, 1520* | 25.013 | 7.85E-12 | 11.105 | 2.86E-10 | 4-0; 4-1; 4-2; 4-3 |
| 4 | L-Glutamic Acid, 2 TMS, 22.75, 1519* | 24.982 | 8.02E-12 | 11.096 | 2.86E-10 | 4-0; 4-1; 4-2; 4-3 |
| 5 | L-Pyroglutamic acid, 2 TMS, 22.76, 1520* | 24.955 | 8.17E-12 | 11.088 | 2.86E-10 | 4-0; 4-1; 4-2; 4-3 |
| 6 | L-Pyroglutamic acid, 2 TMS, 22.76, 1520* | 24.894 | 8.53E-12 | 11.069 | 2.86E-10 | 4-0; 4-1; 4-2; 4-3 |
| 7 | L-Glutamic acid, 2 TMS, 22.7, 1519* | 24.124 | 1.47E-11 | 10.834 | 4.21E-10 | 4-0; 4-1; 4-2; 4-3 |
| 8 | L-Leucine, 2 TMS, 16.74, 1274 | 18.995 | 7.35E-10 | 9.1339 | 1.85E-08 | 4-0; 4-1; 3-2; 4-2; 4-3 |
| 9 | Mix J Unknown 3, 21.95, 1484_malic acid-like | 17.678 | 2.21E-09 | 8.6561 | 4.93E-08 | 4-0; 4-1; 4-2; 4-3 |
| 10 | Unknown_38.802, 2301, m/z387-D-mannose-6-phosphate-like | 16.69 | 5.18E-09 | 8.2853 | 1.04E-07 | 4-0; 4-1; 4-2; 4-3 |
| 11 | Putrescine, 4 TMS, 27.09, 1739 | 14.721 | 3.09E-08 | 7.5102 | 5.64E-07 | 4-0; 4-1; 4-2; 4-3 |
| 12 | Glutamic acid, 3 TMS, 24.79, 1623 | 14.59 | 3.49E-08 | 7.4571 | 5.85E-07 | 4-0; 4-1; 4-2; 4-3 |
| 13 | Putrescine, 4 TMS, 27.09, 1739* | 14.459 | 3.95E-08 | 7.4034 | 5.97E-07 | 4-0; 4-1; 4-2; 4-3 |
| 14 | D-Fructose-6-phosphate 6TMS, MEOX, 36.72, 2300-putative.2 | 14.404 | 4.16E-08 | 7.3811 | 5.97E-07 | 4-0; 4-1; 4-2; 4-3 |
| 15 | Putrescine, 4 TMS, 27.09, 1739* | 14.238 | 4.87E-08 | 7.3127 | 6.52E-07 | 4-0; 4-1; 4-2; 4-3 |
| 16 | Citric acid, 4 TMS, 28.69, 1817 | 13.76 | 7.70E-08 | 7.1136 | 9.67E-07 | 4-0; 4-1; 4-2; 4-3 |
| 17 | Fumaric acid, 2 TMS, 18.29, 1357 | 13.3 | 1.20E-07 | 6.9193 | 1.42E-06 | 4-0; 4-1; 4-2; 4-3 |
| 18 | Glutamic acid, 3 TMS, 24.79, 1623* | 13.175 | 1.36E-07 | 6.8657 | 1.45E-06 | 4-0; 4-1; 4-2; 4-3 |
| 19 | Glutamic acid, 3 TMS, 24.79, 1623* | 13.17 | 1.37E-07 | 6.8638 | 1.45E-06 | 4-0; 4-1; 4-2; 4-3 |
| 20 | D-Glucose-6-phosphate, 6 TMS, MEOX, 37.23, 2332 | 12.357 | 3.08E-07 | 6.5111 | 3.10E-06 | 4-0; 4-1; 4-2; 4-3 |
| 21 | Putrescine, x TMS, 22.45, 1506_putative | 12.044 | 4.24E-07 | 6.3725 | 4.06E-06 | 4-0; 4-1; 4-2; 4-3 |
| 22 | Unknown_21.778, 1476, m/z234_arabino-hexos-2-ulose-like | 11.907 | 4.88E-07 | 6.3114 | 4.26E-06 | 4-0; 4-1; 4-2; 4-3 |
| 23 | D-Fructose-6-phosphate 6TMS, MEOX, 36.72, 2300-putative.1 | 11.889 | 4.97E-07 | 6.3033 | 4.26E-06 | 4-0; 4-1; 4-2; 4-3 |
| 24 | D-Glucose-6-phosphate, 6 TMS, MEOX, 36.95, 2312 | 11.867 | 5.09E-07 | 6.2932 | 4.26E-06 | 4-0; 4-1; 4-2; 4-3 |
| 25 | Unknown_40.226, 2546, m/z387-glucose-6-phosphate-like | 11.039 | 1.21E-06 | 5.9169 | 9.74E-06 | 4-0; 4-1; 4-2; 4-3 |
| 26 | g-Aminobutyric acid, 3 TMS, 22.9, 1526 | 9.6506 | 5.51E-06 | 5.2591 | 4.26E-05 | 4-0; 4-1; 3-2; 4-2 |
| 27 | Creatine. x TMS, 23.39, 1551 | 9.242 | 8.73E-06 | 5.0589 | 6.32E-05 | 4-0; 4-1; 4-2; 4-3 |
| 28 | L-Lysine, x TMS, 29.32, 1852 | 9.2345 | 8.81E-06 | 5.0552 | 6.32E-05 | 4-0; 4-1; 4-2; 4-3 |
| 29 | Unknown_23.930, 1578, m/z129 | 9.1519 | 9.68E-06 | 5.0143 | 6.71E-05 | 4-0; 2-1; 3-1; 4-1; 4-2; 4-3 |
| 30 | a-Ketoglutaric acid, x TMS, 23.95, 1578 | 8.9376 | 1.24E-05 | 4.9077 | 8.29E-05 | 4-0; 4-1; 4-2; 4-3 |
| 31 | Putrescine, 4 TMS, 27.09, 1739* | 8.2195 | 2.86E-05 | 4.5441 | 0.000185 | 4-0; 4-1; 4-2; 4-3 |
| 32 | D-Fructose-6-phosphate 6TMS, MEOX, 36.72, 2300-putative | 7.9135 | 4.11E-05 | 4.386 | 0.000258 | 4-0; 4-1; 4-2; 4-3 |
| 33 | Mix F Unknown 1, 19.28, 1377 | 7.6888 | 5.39E-05 | 4.2688 | 0.000328 | 4-0; 4-1; 4-2; 4-3 |
| 34 | Unknown_31.554, 1971, m/z191-glucose, pentakis-O-TMS-like_Mix A Unknown 11, 31.54, 1971 | 7.3432 | 8.20E-05 | 4.0863 | 0.000485 | 4-0; 4-1; 4-2; 4-3 |
| 35 | 5-Hydroxyindole-3-acetic acid, 3 TMS, 35.49, 2212 | 7.1701 | 0.000101 | 3.9941 | 0.000582 | 4-0; 4-1; 4-2; 4-3 |
| 36 | Unknown_39.306, 2480, m/z315-D-fructose-6-phosphate-like | 6.8186 | 0.000157 | 3.8048 | 0.000875 | 4-0; 4-1; 4-2; 4-3 |
| 37 | Unknown_35.122, 2184, m/z357 | 6.7488 | 0.000171 | 3.7669 | 0.000929 | 4-0; 4-1; 4-2; 4-3 |
| 38 | Unknown_35.898, 2236, m/z188-putative phosphoric acid | 6.0927 | 0.000393 | 3.4055 | 0.002 | 4-0; 4-1; 4-2; 4-3 |
| 39 | Unknown_22.674, 1515, m/z 176 | 6.0829 | 0.000398 | 3.4001 | 0.002 | 4-0; 4-1; 4-2; 4-3 |
| 40 | Unknown_22.674, 1515, m/z 176.1 | 6.0829 | 0.000398 | 3.4001 | 0.002 | 4-0; 4-1; 4-2; 4-3 |
| 41 | Putrescine, 4 TMS, 27.13, 1738* | 6.0287 | 0.000427 | 3.3698 | 0.002092 | 4-0; 4-1; 4-2; 4-3 |
| 42 | Ascorbic acid, x TMS, 29.19, 1844 | 5.9721 | 0.000459 | 3.3382 | 0.002197 | 4-0; 4-1; 4-2; 4-3 |
| 43 | artifact.1 | 5.7875 | 0.000583 | 3.2343 | 0.002725 | 4-0; 4-1; 4-2 |
| 44 | Serine, 3 TMS, 18.98, 1363 | 5.5908 | 0.000753 | 3.1229 | 0.003442 | 3-0; 4-0; 4-1; 4-2 |
| 45 | Unknown_23.546, 1558, m/z 292-threonic acid-like | 5.2988 | 0.001107 | 2.956 | 0.004861 | 4-0; 4-1; 4-3 |
| 46 | Unknown_34.074, 2121, m/z319-galactose-like | 5.2886 | 0.001122 | 2.9502 | 0.004861 | 4-1; 4-2 |
| 47 | Unknown_38.338, 2411, m/z217-uridine-3TMS-like | 5.2786 | 0.001137 | 2.9444 | 0.004861 | 4-0; 4-2; 4-3 |
| 48 | Unknown_26.066, 1685, m/z159 | 5.2071 | 0.00125 | 2.9032 | 0.005233 | 3-2; 4-2 |
| 49 | Sucrose, 8 TMS, 41.32, 2630.1 | 5.1099 | 0.001422 | 2.8471 | 0.005833 | 1-0; 2-0; 3-1; 3-2 |
| 50 | Unknown_42.354, 2698, m/z289 | 5.0845 | 0.001471 | 2.8324 | 0.005914 | 2-1; 4-2 |
| 51 | DL-Ornithine, 3 TMS, 24.71, 1623 | 4.9852 | 0.00168 | 2.7748 | 0.00662 | 4-0; 4-1; 4-3 |
| 52 | Unknown_37.130, 2142, m/z315-myo-inositol-2-phosphate-like.1 | 4.9388 | 0.001787 | 2.7478 | 0.006909 | 4-0; 4-1; 4-2; 4-3 |
| 53 | Unknown_24.994, 1631, m/z174 | 4.8803 | 0.001933 | 2.7137 | 0.007197 | 4-0; 4-1; 4-2; 4-3 |
| 54 | Unknown_24.994, 1631, m/z174.1 | 4.8803 | 0.001933 | 2.7137 | 0.007197 | 4-0; 4-1; 4-2; 4-3 |
| 55 | Sugar alcohol, 6 TMS, 30.6, 1915* | 4.7643 | 0.00226 | 2.6459 | 0.00826 | 4-0; 4-2; 4-3 |
| 56 | Unknown_17.908, 1332, m/z247_succinic acid-like | 4.6699 | 0.002567 | 2.5905 | 0.009215 | 4-0; 4-2; 4-3 |
| 57 | Unknown, 20.834, 1437, m/z 158 | 4.6256 | 0.002726 | 2.5644 | 0.009613 | 4-0; 4-2; 4-3 |
| 58 | Unknown_37.130, 2142, m/z315-myo-inositol-2-phosphate-like | 4.5922 | 0.002852 | 2.5448 | 0.009884 | 4-0; 4-1; 4-2; 4-3 |
| 59 | Unknown_42.674, 2721, m/z361 | 4.4853 | 0.003298 | 2.4817 | 0.011165 | 2-0; 4-2 |
| 60 | Unknown_42.354, 2698, m/z289.1 | 4.4777 | 0.003333 | 2.4772 | 0.011165 | 4-1; 4-2 |
| 61 | Unknown_21.714, 1437, m/z 68_hydroxy proline-like | 4.4363 | 0.003526 | 2.4527 | 0.01162 | 4-0; 4-3 |
| 62 | Cellobiose, x TMS, 42.19, 2962 | 4.4192 | 0.00361 | 2.4425 | 0.011702 | 3-2; 4-2 |
| 63 | Unknown_39.098, 2465, m/z285, m/z285 | 4.3756 | 0.003831 | 2.4167 | 0.012223 | 1-0; 4-0; 3-1; 4-3 |
| 64 | Pantothenic acid, O,O,O-TMS-putative | 4.2673 | 0.004443 | 2.3523 | 0.013954 | 4-0; 4-2; 4-3 |
| 65 | Adenine, 2 TMS, 29.74, 1869 | 4.1181 | 0.005455 | 2.2632 | 0.016867 | 4-0; 4-1; 4-2; 4-3 |
| 66 | Unknown_34.17, 2127, m/z319-galactose-like | 4.0619 | 0.005894 | 2.2296 | 0.017949 | 4-2 |
| 67 | D-Fructose-1-phosphate, x TMS, 36.58, 2290 | 3.9434 | 0.006943 | 2.1584 | 0.02083 | 4-0; 4-1; 4-2 |
| 68 | D--Turanose, 7 TMS, 42.29, 2702 | 3.8839 | 0.00754 | 2.1226 | 0.022287 | 4-2 |
| 69 | D--Galactose, 5 TMS, MEOX, 29.94, 1880 | 3.7762 | 0.008757 | 2.0577 | 0.025509 | 4-0; 4-1 |
| 70 | Unknown_20.426, 1421, m/z 172 | 3.7054 | 0.009665 | 2.0148 | 0.027693 | 3-0; 3-2 |
| 71 | Unknown_21.482, 1464, m/z 204 | 3.6967 | 0.009782 | 2.0096 | 0.027693 |  |
| 72 | Unknown_22.370, 1500, m/z 115 | 3.6734 | 0.010106 | 1.9954 | 0.028211 | 4-0; 4-2 |
| 73 | Ribulose-5-phosphate, 5 TMS, MEOX, 33.83, 2111 | 3.4933 | 0.012999 | 1.8861 | 0.035792 | 4-1; 4-2; 4-3 |
| 74 | Unknown_20.61, 1428, m/z 158_tentative glycine | 3.4479 | 0.013853 | 1.8585 | 0.037627 | 3-1 |
| 75 | Unknown_44.002, 2820, m/z131 | 3.3212 | 0.016551 | 1.7812 | 0.044357 | 4-0 |
| 76 | Mix A Unknown 12, 32.38, 2022 | 3.2573 | 0.01811 | 1.7421 | 0.047897 | 4-2 |
| 77 | Glycine, 3 TMS, 17.63, 1308 | 3.2372 | 0.01863 | 1.7298 | 0.048096 | 4-0 |
| 78 | D-sorbitol 13C6 m/z 323 | 3.227 | 0.018899 | 1.7236 | 0.048096 | 4-3 |
| 79 | D-sorbitol 13C6 m/z_323 | 3.2268 | 0.018903 | 1.7235 | 0.048096 | 4-3 |
|  |  |  |  |  |  |  |

*Additional quantitation calculated from lesser abundant ion, to account for MS detector saturation

#### Table S5. Pattern searching template matching approach (Groups 0-4 in order) on liver samples to determine metabolites with significant comparisons between sampling periods (only showing metabolites with FDR < 0.05).

| # | Metabolite name | correlation | t-stat | p-value | FDR |
| --- | --- | --- | --- | --- | --- |
| 1 | L-Glutamic Acid, 2 TMS, 22.75, 1519* | -0.6347 | -6.2551 | 5.16E-08 | 2.50E-06 |
| 2 | L-Pyroglutamic acid, 2 TMS, 22.76, 1520* | -0.63459 | -6.2534 | 5.19E-08 | 2.50E-06 |
| 3 | L-Pyroglutamic acid, 2 TMS, 22.76, 1520* | -0.63403 | -6.2441 | 5.38E-08 | 2.50E-06 |
| 4 | Unknown, x TMS, 22.73, 1520* | -0.63388 | -6.2417 | 5.43E-08 | 2.50E-06 |
| 5 | L-Glutamic acid, 2 TMS, 22.7, 1519* | -0.6317 | -6.2059 | 6.22E-08 | 2.50E-06 |
| 6 | Putrescine, x TMS, 22.45, 1506_putative | 0.61232 | 5.8983 | 2.00E-07 | 6.71E-06 |
| 7 | L-Glutamic acid, 2 TMS, 22.7, 1519* | -0.60745 | -5.8238 | 2.66E-07 | 7.63E-06 |
| 8 | L-Isoleucine, 2 TMS, 17.32, 1295 | 0.57373 | 5.3348 | 1.65E-06 | 4.15E-05 |
| 9 | L-Leucine, 2 TMS, 16.74, 1274 | 0.57127 | 5.3008 | 1.87E-06 | 4.18E-05 |
| 10 | Putrescine, 4 TMS, 27.09, 1739 | 0.56502 | 5.2154 | 2.56E-06 | 5.09E-05 |
| 11 | Putrescine, 4 TMS, 27.09, 1739* | 0.56335 | 5.1927 | 2.78E-06 | 5.09E-05 |
| 12 | Putrescine, 4 TMS, 27.09, 1739* | 0.5603 | 5.1518 | 3.23E-06 | 5.42E-05 |
| 13 | Mix F Unknown 1, 19.28, 1377 | -0.55072 | -5.0248 | 5.14E-06 | 7.94E-05 |
| 14 | Unknown_21.778, 1476, m/z234_arabino-hexos-2-ulose-like | -0.52525 | -4.7009 | 1.64E-05 | 0.000236 |
| 15 | g-Aminobutyric acid, 3 TMS, 22.9, 1526 | -0.51611 | -4.5891 | 2.43E-05 | 0.000315 |
| 16 | Unknown_23.930, 1578, m/z129 | -0.51538 | -4.5802 | 2.51E-05 | 0.000315 |
| 17 | Putrescine, 4 TMS, 27.09, 1739* | 0.50103 | 4.4091 | 4.55E-05 | 0.000538 |
| 18 | Glutamic acid, 3 TMS, 24.79, 1623 | -0.49642 | -4.3552 | 5.48E-05 | 0.000612 |
| 19 | Serine, 3 TMS, 18.98, 1363 | 0.49314 | 4.317 | 6.25E-05 | 0.000661 |
| 20 | Creatine. x TMS, 23.39, 1551 | 0.48784 | 4.2561 | 7.70E-05 | 0.000752 |
| 21 | Citric acid, 4 TMS, 28.69, 1817 | -0.4873 | -4.2499 | 7.86E-05 | 0.000752 |
| 22 | Mix J Unknown 3, 21.95, 1484_malic acid-like | -0.485 | -4.2237 | 8.59E-05 | 0.000785 |
| 23 | Glutamic acid, 3 TMS, 24.79, 1623* | -0.4782 | -4.1467 | 0.000111 | 0.000934 |
| 24 | Fumaric acid, 2 TMS, 18.29, 1357 | -0.47769 | -4.141 | 0.000114 | 0.000934 |
| 25 | Glutamic acid, 3 TMS, 24.79, 1623* | -0.47708 | -4.1342 | 0.000116 | 0.000934 |
| 26 | Unknown_35.122, 2184, m/z357 | -0.4706 | -4.0619 | 0.000148 | 0.001144 |
| 27 | Unknown_38.802, 2301, m/z387-D-mannose-6-phosphate-like | -0.46912 | -4.0455 | 0.000156 | 0.001163 |
| 28 | Unknown_23.546, 1558, m/z 292-threonic acid-like | -0.46165 | -3.9634 | 0.000205 | 0.001472 |
| 29 | 5-Hydroxyindole-3-acetic acid, 3 TMS, 35.49, 2212 | 0.45911 | 3.9358 | 0.000225 | 0.001556 |
| 30 | D-Fructose-6-phosphate 6TMS, MEOX, 36.72, 2300-putative.2 | -0.44874 | -3.8241 | 0.000323 | 0.002165 |
| 31 | a-Ketoglutaric acid, x TMS, 23.95, 1578 | -0.44337 | -3.7671 | 0.000388 | 0.002517 |
| 32 | D-Fructose-6-phosphate 6TMS, MEOX, 36.72, 2300-putative | -0.43669 | -3.6968 | 0.000486 | 0.003008 |
| 33 | L-Lysine, x TMS, 29.32, 1852 | 0.4358 | 3.6875 | 0.000501 | 0.003008 |
| 34 | Glycine, 3 TMS, 17.63, 1308 | 0.43531 | 3.6824 | 0.000509 | 0.003008 |
| 35 | Unknown_40.226, 2546, m/z387-glucose-6-phosphate-like | -0.4314 | -3.6418 | 0.000579 | 0.003324 |
| 36 | D-Glucose-6-phosphate, 6 TMS, MEOX, 37.23, 2332 | -0.41173 | -3.4408 | 0.001082 | 0.006038 |
| 37 | D-Fructose-6-phosphate 6TMS, MEOX, 36.72, 2300-putative.1 | -0.40876 | -3.411 | 0.001185 | 0.006332 |
| 38 | D-Glucose-6-phosphate, 6 TMS, MEOX, 36.95, 2312 | -0.40842 | -3.4076 | 0.001197 | 0.006332 |
| 39 | D-Fructose-1-phosphate, x TMS, 36.58, 2290 | -0.39462 | -3.2708 | 0.001808 | 0.009318 |
| 40 | D--Galactose, 5 TMS, MEOX, 29.94, 1880 | -0.38718 | -3.1981 | 0.002242 | 0.011265 |
| 41 | Unknown_22.674, 1515, m/z 176 | 0.38488 | 3.1758 | 0.002394 | 0.011411 |
| 42 | Unknown_22.674, 1515, m/z 176.1 | 0.38488 | 3.1758 | 0.002394 | 0.011411 |
| 43 | Putrescine, 4 TMS, 27.13, 1738* | 0.38419 | 3.1691 | 0.002441 | 0.011411 |
| 44 | Unknown_22.370, 1500, m/z 115 | 0.37194 | 3.0515 | 0.003431 | 0.015675 |
| 45 | Unknown_26.066, 1685, m/z159 | -0.36557 | -2.9912 | 0.004075 | 0.017977 |
| 46 | Unknown_44.002, 2820, m/z131 | 0.36474 | 2.9833 | 0.004167 | 0.017977 |
| 47 | Pantothenic acid, O,O,O-TMS-putative | 0.36441 | 2.9802 | 0.004204 | 0.017977 |
| 48 | Unknown_37.130, 2142, m/z315-myo-inositol-2-phosphate-like | -0.36136 | -2.9515 | 0.004557 | 0.019083 |
| 49 | Mix N Unknown 1, 22.98, 1532 | -0.35657 | -2.9066 | 0.005167 | 0.021197 |
| 50 | L-Phenylalanine, 1 TMS, 23.37, 1550 | 0.3533 | 2.8761 | 0.005623 | 0.022606 |
| 51 | DL-Ornithine, 3 TMS, 24.71, 1623 | 0.3453 | 2.802 | 0.006892 | 0.027161 |
| 52 | Unknown_31.554, 1971, m/z191-glucose, pentakis-O-TMS-like_Mix A Unknown 11, 31.54, 1971 | -0.32901 | -2.6534 | 0.010265 | 0.039676 |
| 53 | Unknown_37.130, 2142, m/z315-myo-inositol-2-phosphate-like.1 | -0.32705 | -2.6357 | 0.010754 | 0.040785 |
| 54 | Unknown_20.426, 1421, m/z 172 | 0.32465 | 2.6141 | 0.011381 | 0.042361 |
| 55 | Unknown_28.530, 1808, m/z 357-ribulose 5-phosphate-like | -0.3226 | -2.5956 | 0.01194 | 0.043635 |
| 56 | artifact.1 | -0.31772 | -2.5519 | 0.013369 | 0.047596 |
| 57 | Unknown_24.994, 1631, m/z174 | -0.31655 | -2.5415 | 0.013734 | 0.047596 |
| 58 | Unknown_24.994, 1631, m/z174.1 | -0.31655 | -2.5415 | 0.013734 | 0.047596 |
| 59 | Unknown_38.338, 2411, m/z217-uridine-3TMS-like | -0.31507 | -2.5282 | 0.014208 | 0.048405 |
| 60 | Unknown, 20.834, 1437, m/z 158 | 0.31355 | 2.5147 | 0.014708 | 0.049271 |
|  |  |  |  |  |  |

*Additional quantitation calculated from lesser abundant ion, to account for MS detector saturation

#### Table S6. Significance Analysis of Microarrays on liver samples to determine metabolites with significant differences between sampling periods (only showing metabolites with FDR < 0.05).

| # | Metabolite name | d.value | stdev | rawp | FDR |
| --- | --- | --- | --- | --- | --- |
| 1 | 5-Hydroxyindole-3-acetic acid, 3 TMS, 35.49, 2212 | 2.0432 | 2.2227 | 0.000149 | 0.003029 |
| 2 | Citric acid, 4 TMS, 28.69, 1817 | 1.9118 | 0.89996 | 0.000249 | 0.003029 |
| 3 | Putrescine, x TMS, 22.45, 1506_putative | 1.8058 | 0.98367 | 0.000249 | 0.003029 |
| 4 | Unknown_38.802, 2301, m/z387-D-mannose-6-phosphate | 1.7695 | 0.66144 | 0.000299 | 0.003029 |
| 5 | Sugar alcohol, 6 TMS, 30.6, 1915* | 1.7392 | 3.2066 | 0.000299 | 0.003029 |
| 6 | Unknown_40.226, 2546, m/z387-glucose-6-phosphate-l | 1.737 | 1.0414 | 0.000299 | 0.003029 |
| 7 | a-Ketoglutaric acid, x TMS, 23.95, 1578 | 1.6852 | 1.2959 | 0.000348 | 0.003029 |
| 8 | D--Galactose, 5 TMS, MEOX, 29.94, 1880 | 1.6133 | 4.1599 | 0.000398 | 0.003029 |
| 9 | Putrescine, 4 TMS, 27.09, 1739 | 1.4993 | 0.63248 | 0.000647 | 0.003029 |
| 10 | Putrescine, 4 TMS, 27.09, 1739* | 1.4962 | 0.64375 | 0.000647 | 0.003029 |
| 11 | artifact.1 | 1.4958 | 1.9439 | 0.000647 | 0.003029 |
| 12 | Putrescine, 4 TMS, 27.09, 1739* | 1.4957 | 0.65465 | 0.000647 | 0.003029 |
| 13 | D-Fructose-6-phosphate 6TMS, MEOX, 36.72, 2300-put | 1.4934 | 0.64513 | 0.000647 | 0.003029 |
| 14 | Creatine. x TMS, 23.39, 1551 | 1.4805 | 1.0639 | 0.000746 | 0.003029 |
| 15 | Unknown_21.778, 1476, m/z234_arabino-hexos-2-ulose | 1.4796 | 0.79133 | 0.000746 | 0.003029 |
| 16 | D-Glucose-6-phosphate, 6 TMS, MEOX, 37.23, 2332 | 1.4495 | 0.74112 | 0.000796 | 0.003029 |
| 17 | D-Fructose-6-phosphate 6TMS, MEOX, 36.72, 2300-put | 1.4431 | 1.2439 | 0.000796 | 0.003029 |
| 18 | Mix J Unknown 3, 21.95, 1484_malic acid-like | 1.4127 | 0.4844 | 0.000896 | 0.003029 |
| 19 | Unknown_35.898, 2236, m/z188-putative phosphoric a | 1.3884 | 1.646 | 0.000945 | 0.003029 |
| 20 | Unknown_23.930, 1578, m/z129 | 1.382 | 0.99198 | 0.000995 | 0.003029 |
| 21 | D-Fructose-6-phosphate 6TMS, MEOX, 36.72, 2300-put | 1.3754 | 0.72958 | 0.000995 | 0.003029 |
| 22 | D-Glucose-6-phosphate, 6 TMS, MEOX, 36.95, 2312 | 1.3737 | 0.73014 | 0.000995 | 0.003029 |
| 23 | Unknown_42.354, 2698, m/z289 | 1.3671 | 2.0512 | 0.000995 | 0.003029 |
| 24 | L-Isoleucine, 2 TMS, 17.32, 1295 | 1.3203 | 0.24918 | 0.001294 | 0.003774 |
| 25 | Unknown_35.122, 2184, m/z357 | 1.3093 | 1.3425 | 0.001393 | 0.003902 |
| 26 | L-Lysine, x TMS, 29.32, 1852 | 1.2717 | 0.89076 | 0.001493 | 0.00402 |
| 27 | Putrescine, 4 TMS, 27.09, 1739* | 1.2537 | 1.0038 | 0.001642 | 0.004258 |
| 28 | Unknown_39.306, 2480, m/z315-D-fructose-6-phosphat | 1.196 | 1.1864 | 0.00199 | 0.004805 |
| 29 | g-Aminobutyric acid, 3 TMS, 22.9, 1526 | 1.1951 | 0.78826 | 0.00199 | 0.004805 |
| 30 | Unknown_31.554, 1971, m/z191-glucose, pentakis-O-T | 1.1732 | 1.0605 | 0.002189 | 0.00511 |
| 31 | Fumaric acid, 2 TMS, 18.29, 1357 | 1.1466 | 0.52618 | 0.002537 | 0.005732 |
| 32 | Putrescine, 4 TMS, 27.13, 1738* | 1.1301 | 1.2867 | 0.002836 | 0.006206 |
| 33 | Mix F Unknown 1, 19.28, 1377 | 1.108 | 0.93904 | 0.003383 | 0.007173 |
| 34 | Unknown_21.714, 1437, m/z 68_hydroxy proline-like | 1.1024 | 1.8443 | 0.003483 | 0.007173 |
| 35 | Sucrose, 8 TMS, 41.32, 2630.1 | 1.0669 | 1.4718 | 0.004179 | 0.008361 |
| 36 | Adenine, 2 TMS, 29.74, 1869 | 1.0055 | 1.8018 | 0.005721 | 0.011043 |
| 37 | D--Turanose, 7 TMS, 42.29, 2702 | 0.99853 | 1.9301 | 0.00607 | 0.011043 |
| 38 | D-sorbitol 13C6 m/z 323 | 0.99199 | 2.4754 | 0.006269 | 0.011043 |
| 39 | D-sorbitol 13C6 m/z 323 | 0.99195 | 2.4754 | 0.006269 | 0.011043 |
| 40 | Ascorbic acid, x TMS, 29.19, 1844 | 0.98118 | 1.0964 | 0.006567 | 0.011043 |
| 41 | Unknown, 20.834, 1437, m/z 158 | 0.97766 | 1.4947 | 0.006667 | 0.011043 |
| 42 | Glutamic acid, 3 TMS, 24.79, 1623 | 0.96087 | 0.3932 | 0.007114 | 0.011043 |
| 43 | Unknown, x TMS, 22.73, 1520* | 0.94729 | 0.21953 | 0.007413 | 0.011043 |
| 44 | L-Glutamic Acid, 2 TMS, 22.75, 1519* | 0.94708 | 0.21977 | 0.007413 | 0.011043 |
| 45 | L-Pyroglutamic acid, 2 TMS, 22.76, 1520* | 0.94644 | 0.21986 | 0.007413 | 0.011043 |
| 46 | L-Glutamic acid, 2 TMS, 22.7, 1519* | 0.94574 | 0.21694 | 0.007512 | 0.011043 |
| 47 | L-Pyroglutamic acid, 2 TMS, 22.76, 1520* | 0.94543 | 0.22018 | 0.007512 | 0.011043 |
| 48 | Unknown_42.354, 2698, m/z289.1 | 0.91666 | 1.4357 | 0.008358 | 0.011043 |
| 49 | Glutamic acid, 3 TMS, 24.79, 1623* | 0.91643 | 0.41696 | 0.008358 | 0.011043 |
| 50 | L-Glutamic acid, 2 TMS, 22.7, 1519* | 0.91642 | 0.22023 | 0.008358 | 0.011043 |
| 51 | Urea, 2 TMS, 16.14, 1249 | 0.91601 | 5.5773 | 0.008358 | 0.011043 |
| 52 | Unknown_34.074, 2121, m/z319-galactose-like | 0.91435 | 1.1658 | 0.008358 | 0.011043 |
| 53 | Glutamic acid, 3 TMS, 24.79, 1623* | 0.91413 | 0.41599 | 0.008358 | 0.011043 |
| 54 | Potential artifact | 0.90972 | 2.3394 | 0.008657 | 0.011226 |
| 55 | Unknown_42.674, 2721, m/z361 | 0.89909 | 1.3983 | 0.008906 | 0.011264 |
| 56 | Cellobiose, x TMS, 42.19, 2962 | 0.88307 | 1.3928 | 0.009552 | 0.011264 |
| 57 | Unknown_38.338, 2411, m/z217-uridine-3TMS-like | 0.88255 | 1.1197 | 0.009552 | 0.011264 |
| 58 | Unknown_40.714, 2581, m/z217.2 | 0.88218 | 2.2595 | 0.009552 | 0.011264 |
| 59 | L-Leucine, 2 TMS, 16.74, 1274 | 0.87608 | 0.26967 | 0.009652 | 0.011264 |
| 60 | Unknown_41.866, 2664, m/z204 | 0.87535 | 2.2045 | 0.009652 | 0.011264 |
| 61 | Unknown_37.130, 2142, m/z315-myo-inositol-2-phosph | 0.86162 | 1.1786 | 0.01005 | 0.011537 |
| 62 | Unknown_37.130, 2142, m/z315-myo-inositol-2-phosph | 0.82217 | 1.2163 | 0.012189 | 0.013767 |
| 63 | Unknown_39.098, 2465, m/z285, m/z285 | 0.81646 | 1.2794 | 0.012537 | 0.013935 |
| 64 | Unknown_26.066, 1685, m/z159 | 0.80431 | 1.0189 | 0.013333 | 0.014589 |
| 65 | Unknown_40.714, 2581, m/z217 | 0.77979 | 2.0669 | 0.014925 | 0.016079 |
| 66 | Unknown_34.17, 2127, m/z319-galactose-like | 0.76934 | 1.3032 | 0.015771 | 0.016733 |
| 67 | Unknown_24.994, 1631, m/z174.1 | 0.74405 | 1.0033 | 0.017413 | 0.017932 |
| 68 | Unknown_24.994, 1631, m/z174 | 0.74405 | 1.0033 | 0.017413 | 0.017932 |
| 69 | Unknown_21.482, 1464, m/z 204 | 0.73094 | 1.3746 | 0.018408 | 0.018682 |
| 70 | likely artifact-glucose-like.1 | 0.71542 | 3.27 | 0.019353 | 0.019088 |
| 71 | likely artifact-glucose-like | 0.71542 | 3.27 | 0.019353 | 0.019088 |
| 72 | Unknown_44.002, 2820, m/z131 | 0.70707 | 1.5085 | 0.01995 | 0.01928 |
| 73 | Unknown_42.354, 2704, m/z217 | 0.70596 | 2.2458 | 0.0201 | 0.01928 |
| 74 | Unknown_43.018, 2746, m/z361 | 0.64884 | 1.9548 | 0.027413 | 0.025941 |
| 75 | Unknown_23.546, 1558, m/z 292-threonic acid-like | 0.6464 | 0.7749 | 0.02791 | 0.026059 |
| 76 | Unknown_40.226, 2546, m/z387-glucose-6-phosphate-l | 0.6413 | 1.6725 | 0.028955 | 0.026513 |
| 77 | DL-Ornithine, 3 TMS, 24.71, 1623 | 0.63937 | 0.82055 | 0.029154 | 0.026513 |
| 78 | Unknown_20.61, 1428, m/z 158_tentative glycine | 0.63469 | 1.2583 | 0.0301 | 0.027022 |
| 79 | L-Threonine, 2 TMS, 17.37, 1298 | 0.6109 | 1.4331 | 0.034279 | 0.030384 |
| 80 | D--Glucose, 5 TMS, MEOX, 30.38, 1902* | 0.59974 | 3.4857 | 0.037114 | 0.032487 |
| 81 | Pantothenic acid, O,O,O-TMS-putative | 0.59327 | 0.9006 | 0.03791 | 0.032587 |
| 82 | D--Galactose, 5 TMS, MEOX, 29.94, 1880* | 0.59156 | 1.5652 | 0.038159 | 0.032587 |
| 83 | L-Methionine, 1 TMS, 20.27, 1416_putative.2 | 0.58642 | 2.0869 | 0.039602 | 0.033221 |
| 84 | Unknown_36.242, 2261, m/z315 | 0.58573 | 1.6287 | 0.039851 | 0.033221 |
| 85 | D-Fructose-1-phosphate, x TMS, 36.58, 2290 | 0.58074 | 0.96322 | 0.040846 | 0.03365 |
| 86 | Unknown_22.370, 1500, m/z 115 | 0.57493 | 1.0349 | 0.042139 | 0.034312 |
| 87 | Unknown m/z_320 | 0.57247 | 2.6771 | 0.042687 | 0.034358 |
| 88 | Ribulose-5-phosphate, 5 TMS, MEOX, 33.83, 2111 | 0.55964 | 1.0639 | 0.045721 | 0.035651 |
| 89 | L-Methionine, 1 TMS, 20.27, 1416_putative.1 | 0.55938 | 2.0833 | 0.045821 | 0.035651 |
| 90 | L-Methionine, 1 TMS, 20.27, 1416_putative | 0.55938 | 2.0833 | 0.045821 | 0.035651 |
| 91 | Unknown_28.530, 1808, m/z 357-ribulose 5-phosphate | 0.54686 | 1.2558 | 0.048905 | 0.037633 |
| 92 | Serine, 3 TMS, 18.98, 1363 | 0.54079 | 0.59725 | 0.050547 | 0.038474 |
| 93 | Serine, 2 TMS, 16.43, 1260 | 0.53174 | 1.7163 | 0.052637 | 0.039633 |
| 94 | Unknown_17.908, 1332, m/z247_succinic acid-like | 0.51785 | 0.69561 | 0.057313 | 0.042696 |
| 95 | Mix N Unknown 1, 22.98, 1532 | 0.51231 | 1.3676 | 0.059453 | 0.043823 |
| 96 | Unknown_40.714, 2581, m/z217.1 | 0.4981 | 1.5385 | 0.064428 | 0.046534 |
| 97 | Unknown_22.674, 1515, m/z 176.1 | 0.49579 | 0.49492 | 0.065124 | 0.046534 |
| 98 | Unknown_22.674, 1515, m/z 176 | 0.49579 | 0.49492 | 0.065124 | 0.046534 |
|  |  |  |  |  |  |

*Additional quantitation calculated from lesser abundant ion, to account for MS detector saturation

#### Table S7. Variable Importance in Projection measures of the top 30 ranked metabolites (sorted by component one) from Partial Least Squares discriminant analysis (PLS-DA) of skin samples, grouping by sample period.

| # | Metabolite name | Comp. 1 | Comp. 2 | Comp. 3 | Comp. 4 | Comp. 5 |
| --- | --- | --- | --- | --- | --- | --- |
| 1 | Unknown_46.866, 3064, m/z169 | 2.4994 | 2.0217 | 1.947 | 1.9865 | 1.9889 |
| 2 | Unknown_46.848, 3063, m/z169 | 2.4383 | 1.9715 | 1.8828 | 1.9364 | 1.9411 |
| 3 | a-Ketoglutaric acid, x TMS, 23.95, 1578 | 2.2876 | 2.0712 | 1.8764 | 1.7926 | 1.7712 |
| 4 | Unknown_21.778, 1476, m/z234_arabino-hexos-2-ulose-like | 2.2527 | 1.8915 | 1.7772 | 1.7374 | 1.6974 |
| 5 | Unknown m/z_320 | 2.2286 | 2.0784 | 2.0537 | 1.965 | 1.9158 |
| 6 | Mix C Unknown 3, 24.71, 1617 | 2.1819 | 1.8926 | 1.6915 | 1.7195 | 1.6831 |
| 7 | Putrescine, x TMS, 22.45, 1506_putative | 2.0726 | 1.7915 | 1.6017 | 1.552 | 1.5135 |
| 8 | Mix N Unknown 1, 22.98, 1532 | 2.0696 | 1.8759 | 1.6945 | 1.6238 | 1.5857 |
| 9 | Unknown_22.954, 1529, m/z 160 | 2.0394 | 1.9038 | 1.7015 | 1.6348 | 1.6028 |
| 10 | Sugar alcohol, 6 TMS, 30.6, 1915* | 2.0275 | 1.9893 | 1.9738 | 1.8942 | 1.8447 |
| 11 | Adenine, 2 TMS, 29.74, 1869 | 2.0262 | 1.7629 | 1.6074 | 1.5345 | 1.4959 |
| 12 | Unknown_23.546, 1558, m/z 292-threonic acid-like | 2.0182 | 1.7336 | 1.5708 | 1.5828 | 1.5817 |
| 13 | L-Threonine, 2 TMS, 17.37, 1298 | 1.99 | 1.6145 | 1.4665 | 1.4107 | 1.4903 |
| 14 | Unknown_26.066, 1685, m/z159 | 1.8796 | 1.5369 | 1.3788 | 1.367 | 1.3315 |
| 15 | L-Threonine, 3 TMS, 19.59, 1387 | 1.775 | 1.6962 | 1.6755 | 1.6159 | 1.5735 |
| 16 | Putrescine, 4 TMS, 27.09, 1739* | 1.7408 | 1.5329 | 1.381 | 1.3202 | 1.2856 |
| 17 | Putrescine, 4 TMS, 27.09, 1739* | 1.7395 | 1.5328 | 1.3806 | 1.3197 | 1.2851 |
| 18 | Putrescine, 4 TMS, 27.09, 1739 | 1.7383 | 1.5319 | 1.3798 | 1.3189 | 1.2843 |
| 19 | Putrescine, 4 TMS, 27.09, 1739* | 1.7383 | 1.5319 | 1.3798 | 1.3189 | 1.2843 |
| 20 | Putrescine, 4 TMS, 27.13, 1738* | 1.7383 | 1.5319 | 1.3798 | 1.3189 | 1.2843 |
| 21 | Serine, 2 TMS, 16.43, 1260 | 1.719 | 1.4037 | 1.3544 | 1.2928 | 1.2919 |
| 22 | L-Lysine, 4 TMS, 30.54, 1915 | 1.7061 | 1.6393 | 1.468 | 1.4386 | 1.4218 |
| 23 | Aspartic acid, 2 TMS, 20.54, 1428 | 1.651 | 1.7132 | 1.5314 | 1.4709 | 1.4345 |
| 24 | Serotonin, x TMS, 39.11, 2470 | 1.6147 | 1.327 | 1.2107 | 1.2087 | 1.2579 |
| 25 | Serotonin, x TMS, 39.11, 2470* | 1.5744 | 1.3108 | 1.2234 | 1.2386 | 1.2972 |
| 26 | D-sorbitol, m/z_319 | 1.5404 | 1.7152 | 1.6329 | 1.5653 | 1.5261 |
| 27 | Mix F Unknown 1, 19.28, 1377 | 1.537 | 1.2447 | 1.2785 | 1.2278 | 1.2131 |
| 28 | Cellobiose, x TMS, 42.19, 2962 | 1.496 | 1.3584 | 1.4037 | 1.3587 | 1.3265 |
| 29 | likely artifact-glucose-like | 1.4713 | 1.2491 | 1.1602 | 1.1091 | 1.08 |
| 30 | likely artifact-glucose-like.1 | 1.4713 | 1.2491 | 1.1602 | 1.1091 | 1.08 |
|  |  |  |  |  |  |  |

*Additional quantitation calculated from lesser abundant ion, to account for MS detector saturation

#### Table S8. Variable Importance in Projection measures of the top 30 ranked metabolites (sorted by component one) from Partial Least Squares discriminant analysis (PLS-DA) of liver samples, grouping by sample period.

| # | Metabolite name | Comp. 1 | Comp. 2 | Comp. 3 | Comp. 4 | Comp. 5 |
| --- | --- | --- | --- | --- | --- | --- |
| 1 | D--Galactose, 5 TMS, MEOX, 29.94, 1880 | 2.6805 | 2.2557 | 2.1351 | 2.2482 | 2.215 |
| 2 | 5-Hydroxyindole-3-acetic acid, 3 TMS, 35.49, 2212 | 2.5384 | 2.3434 | 2.1648 | 2.0909 | 2.0384 |
| 3 | Putrescine, x TMS, 22.45, 1506_putative | 2.5008 | 2.3112 | 2.1323 | 2.0359 | 1.9849 |
| 4 | Mix F Unknown 1, 19.28, 1377 | 2.0035 | 1.7821 | 1.6794 | 1.6065 | 1.5659 |
| 5 | Unknown_35.122, 2184, m/z357 | 2.0017 | 1.6807 | 1.6269 | 1.5706 | 1.5684 |
| 6 | Unknown_23.930, 1578, m/z129 | 1.9918 | 1.7568 | 1.6196 | 1.5418 | 1.506 |
| 7 | Citric acid, 4 TMS, 28.69, 1817 | 1.966 | 1.7204 | 1.6899 | 1.6098 | 1.5693 |
| 8 | Creatine. x TMS, 23.39, 1551 | 1.9563 | 1.6529 | 1.5239 | 1.5347 | 1.5037 |
| 9 | a-Ketoglutaric acid, x TMS, 23.95, 1578 | 1.9493 | 1.6427 | 1.5832 | 1.5364 | 1.5011 |
| 10 | Putrescine, 4 TMS, 27.09, 1739* | 1.9465 | 1.6602 | 1.5373 | 1.4705 | 1.4339 |
| 11 | Putrescine, 4 TMS, 27.09, 1739* | 1.9447 | 1.6562 | 1.5338 | 1.4668 | 1.4304 |
| 12 | Putrescine, 4 TMS, 27.09, 1739 | 1.9441 | 1.6594 | 1.5368 | 1.4702 | 1.4335 |
| 13 | Unknown_21.778, 1476, m/z234_arabino-hexos-2-ulose-like | 1.919 | 1.6492 | 1.5319 | 1.4728 | 1.4358 |
| 14 | Putrescine, 4 TMS, 27.09, 1739* | 1.9077 | 1.6376 | 1.5393 | 1.4714 | 1.4355 |
| 15 | D-Fructose-6-phosphate 6TMS, MEOX, 36.72, 2300-putative | 1.8381 | 1.5624 | 1.4579 | 1.4021 | 1.3715 |
| 16 | g-Aminobutyric acid, 3 TMS, 22.9, 1526 | 1.7973 | 1.7139 | 1.6245 | 1.551 | 1.5117 |
| 17 | Unknown_40.226, 2546, m/z387-glucose-6-phosphate-like | 1.7773 | 1.4962 | 1.382 | 1.3304 | 1.3015 |
| 18 | Unknown_38.802, 2301, m/z387-D-mannose-6-phosphate-like | 1.7068 | 1.4858 | 1.3794 | 1.3186 | 1.2864 |
| 19 | L-Lysine, x TMS, 29.32, 1852 | 1.5988 | 1.3575 | 1.3702 | 1.3075 | 1.2743 |
| 20 | artifact.1 | 1.5876 | 1.477 | 1.4457 | 1.3999 | 1.388 |
| 21 | Putrescine, 4 TMS, 27.13, 1738* | 1.5715 | 1.3305 | 1.3208 | 1.2672 | 1.2384 |
| 22 | L-Isoleucine, 2 TMS, 17.32, 1295 | 1.5512 | 1.3179 | 1.215 | 1.1578 | 1.1348 |
| 23 | D-Fructose-6-phosphate 6TMS, MEOX, 36.72, 2300-putative.2 | 1.5506 | 1.4024 | 1.3163 | 1.2531 | 1.2232 |
| 24 | Mix J Unknown 3, 21.95, 1484_malic acid-like | 1.5344 | 1.2915 | 1.2925 | 1.2304 | 1.2026 |
| 25 | Urea, 2 TMS, 16.14, 1249 | 1.5188 | 1.9509 | 1.8379 | 1.7561 | 1.7758 |
| 26 | Unknown_53.63, 3702, m/z311-stearic acid-like | 1.5134 | 1.411 | 1.307 | 1.4787 | 1.4913 |
| 27 | L-Glutamic Acid, 2 TMS, 22.75, 1519* | 1.5014 | 1.3044 | 1.2112 | 1.1711 | 1.143 |
| 28 | L-Pyroglutamic acid, 2 TMS, 22.76, 1520* | 1.501 | 1.3039 | 1.2107 | 1.1706 | 1.1426 |
| 29 | Unknown_44.002, 2820, m/z131 | 1.5008 | 1.3687 | 1.2896 | 1.2284 | 1.2054 |
| 30 | L-Pyroglutamic acid, 2 TMS, 22.76, 1520* | 1.4995 | 1.3024 | 1.2095 | 1.1699 | 1.142 |
|  |  |  |  |  |  |  |

*Additional quantitation calculated from lesser abundant ion, to account for MS detector saturation

#### Table S9. One-way ANOVA Tukey's honestly significant difference post-hoc tests on skin samples to determine significant comparisons between populations (only showing metabolites with FDR < 0.05).

| # | Metabolite name | f.value | p.value | -log10(p) | FDR | Tukey's HSD |
| --- | --- | --- | --- | --- | --- | --- |
| 1 | Pantothenic acid, O,O,O-TMS-putative | 11.23 | 9.98E-05 | 4.0009 | 0.012106 | Greymare-Eucumbene; Kiandra-Eucumbene |
| 2 | Unknown_40.714, 2581, m/z217.2 | 10.969 | 0.000119 | 3.9235 | 0.012106 | Greymare-Eucumbene; Kiandra-Eucumbene |
| 3 | Myo-Inositol, 6 TMS, 33.38, 2081 | 9.8926 | 0.000253 | 3.5975 | 0.017097 | Kiandra-Eucumbene; Kiandra-Greymare |
| 4 | Unknown_35.234, 2190, m/z200-spermine-like | 8.6189 | 0.000634 | 3.1982 | 0.026147 | Greymare-Eucumbene; Kiandra-Greymare |
| 5 | Unknown_40.714, 2581, m/z217 | 8.508 | 0.000688 | 3.1627 | 0.026147 | Kiandra-Eucumbene |
| 6 | Unknown_24.994, 1631, m/z174 | 8.1429 | 0.000902 | 3.045 | 0.026147 | Greymare-Eucumbene; Kiandra-Eucumbene |
| 7 | Unknown_24.994, 1631, m/z174.1 | 8.1429 | 0.000902 | 3.045 | 0.026147 | Greymare-Eucumbene; Kiandra-Eucumbene |
| 8 | L-Leucine, 2 TMS, 16.74, 1274 | 7.8364 | 0.001135 | 2.9451 | 0.026777 | Kiandra-Eucumbene; Kiandra-Greymare |
| 9 | Unknown_22.370, 1500, m/z 115 | 7.6866 | 0.001271 | 2.896 | 0.026777 | Kiandra-Eucumbene; Kiandra-Greymare |
| 10 | L-Isoleucine, 2 TMS, 17.32, 1295 | 7.6374 | 0.001319 | 2.8797 | 0.026777 | Kiandra-Eucumbene |
| 11 | Creatine. x TMS, 23.39, 1551 | 6.7832 | 0.002544 | 2.5945 | 0.046951 | Kiandra-Eucumbene; Kiandra-Greymare |
|  |  |  |  |  |  |  |

#### Table S10. Pattern searching template matching approach (Eucumbene-Grey Mare-Kiandra) on skin samples to determine analytes with significant comparisons between populations (only showing metabolites with FDR < 0.05).

| # | Metabolite name | correlation | t-stat | p-value | FDR |
| --- | --- | --- | --- | --- | --- |
| 1 | Pantothenic acid, O,O,O-TMS-putative | -0.51381 | -4.1924 | 0.000115 | 0.010965 |
| 2 | Unknown_40.714, 2581, m/z217 | 0.51114 | 4.1628 | 0.000127 | 0.010965 |
| 3 | Unknown_40.714, 2581, m/z217.2 | 0.50316 | 4.0756 | 0.000168 | 0.010965 |
| 4 | L-Isoleucine, 2 TMS, 17.32, 1295 | 0.49005 | 3.9353 | 0.000262 | 0.010965 |
| 5 | Unknown_22.370, 1500, m/z 115 | 0.48281 | 3.8592 | 0.000333 | 0.010965 |
| 6 | L-Leucine, 2 TMS, 16.74, 1274 | 0.48099 | 3.8403 | 0.000353 | 0.010965 |
| 7 | Unknown_24.994, 1631, m/z174 | -0.47472 | -3.7756 | 0.000432 | 0.010965 |
| 8 | Unknown_24.994, 1631, m/z174.1 | -0.47472 | -3.7756 | 0.000432 | 0.010965 |
| 9 | Creatine. x TMS, 23.39, 1551 | 0.45692 | 3.5958 | 0.00075 | 0.016922 |
| 10 | Unknown_22.674, 1515, m/z 176 | 0.4307 | 3.3406 | 0.001605 | 0.0295 |
| 11 | Unknown_22.674, 1515, m/z 176.1 | 0.4307 | 3.3406 | 0.001605 | 0.0295 |
| 12 | Unknown_22.954, 1529, m/z 160 | -0.42772 | -3.3123 | 0.001744 | 0.0295 |
| 13 | D---Ribose, 4 TMS, MEOX, 25.89, 1678 | 0.42058 | 3.245 | 0.002119 | 0.033096 |
|  |  |  |  |  |  |

#### Table S11. Significance Analysis of Microarrays on skin samples to determine metabolites with significant differences between populations (only showing metabolites with FDR < 0.05).

| # | Metabolite name | d.value | stdev | rawp | FDR |
| --- | --- | --- | --- | --- | --- |
| 1 | Unknown_40.714, 2581, m/z217.2 | 8.3493 | 1.9395 | 4.93E-05 | 0.002488 |
| 2 | Pantothenic acid, O,O,O-TMS-putative | 6.7497 | 0.91687 | 4.93E-05 | 0.002488 |
| 3 | Unknown_35.234, 2190, m/z200-sperimine-like | 6.4644 | 1.8261 | 4.93E-05 | 0.002488 |
| 4 | Unknown_24.994, 1631, m/z174.1 | 5.8266 | 1.531 | 0.000197 | 0.00597 |
| 5 | Unknown_24.994, 1631, m/z174 | 5.8266 | 1.531 | 0.000197 | 0.00597 |
| 6 | Myo-Inositol, 6 TMS, 33.38, 2081 | 5.1013 | 0.64798 | 0.000788 | 0.0199 |
| 7 | Unknown_40.714, 2581, m/z217 | 4.778 | 0.77959 | 0.00133 | 0.028783 |
| 8 | Unknown_22.954, 1529, m/z 160 | 4.4508 | 2.1956 | 0.001773 | 0.033581 |
| 9 | Mix C Unknown 3, 24.71, 1617 | 4.3151 | 1.6083 | 0.002217 | 0.036566 |
| 10 | Creatine. x TMS, 23.39, 1551 | 4.2274 | 1.0067 | 0.002414 | 0.036566 |
| 11 | L-Leucine, 2 TMS, 16.74, 1274 | 4.0764 | 0.65983 | 0.003153 | 0.039608 |
| 12 | Unknown_22.370, 1500, m/z 115 | 3.9797 | 0.65341 | 0.003399 | 0.039608 |
| 13 | Unknown_29.866, 1875, m/z103 | 3.9666 | 1.1285 | 0.003399 | 0.039608 |
|  |  |  |  |  |  |

#### Table S12. Variable Importance in Projection measures of the top 30 ranked metabolites (sorted by component one) from Partial Least Squares discriminant analysis (PLS-DA) of skin samples, grouping by population.

| # | Metabolite name | Comp. 1 | Comp. 2 | Comp. 3 | Comp. 4 | Comp. 5 |
| --- | --- | --- | --- | --- | --- | --- |
| 1 | Unknown_40.714, 2581, m/z217.2 | 3.0052 | 2.8877 | 2.57 | 2.5485 | 2.538 |
| 2 | Unknown_22.954, 1529, m/z 160 | 2.5043 | 2.0915 | 1.8182 | 1.7643 | 1.7352 |
| 3 | Unknown_24.994, 1631, m/z174 | 2.4151 | 2.118 | 1.845 | 1.8657 | 1.8344 |
| 4 | Unknown_24.994, 1631, m/z174.1 | 2.4151 | 2.118 | 1.845 | 1.8657 | 1.8344 |
| 5 | Unknown_34.202, 2129, m/z352-putative guanine.1 | 2.2968 | 1.8563 | 1.6187 | 1.7175 | 1.6807 |
| 6 | Unknown_39.602, 2501, m/z174-serotonin-like | 2.2372 | 1.77 | 1.6199 | 1.5798 | 1.5761 |
| 7 | Pantothenic acid, O,O,O-TMS-putative | 2.1179 | 1.6976 | 1.5333 | 1.4934 | 1.4794 |
| 8 | D-sorbitol m/z_319 | 2.043 | 1.8936 | 1.6817 | 1.6318 | 1.615 |
| 9 | Unknown_34.202, 2129, m/z352-putative guanine | 2.0061 | 2.0515 | 1.8292 | 1.7899 | 1.7667 |
| 10 | Aspartic acid, 2 TMS, 20.54, 1428 | 1.9701 | 1.7977 | 1.5621 | 1.5233 | 1.4912 |
| 11 | L-Threonine, 2 TMS, 17.37, 1298 | 1.8778 | 1.5012 | 1.3676 | 1.3282 | 1.3314 |
| 12 | Unknown_40.714, 2581, m/z217 | 1.8662 | 2.0586 | 1.7943 | 1.7413 | 1.7132 |
| 13 | Creatine. x TMS, 23.39, 1551 | 1.8447 | 1.744 | 1.5235 | 1.4997 | 1.4681 |
| 14 | D-sorbitol m/z_320 | 1.8389 | 2.1021 | 1.8434 | 1.7892 | 1.7597 |
| 15 | Mix A Unknown 12, 32.38, 2022 | 1.7883 | 1.4173 | 1.2676 | 1.24 | 1.2445 |
| 16 | Guanine manual* | 1.7444 | 1.3837 | 1.2097 | 1.3327 | 1.3168 |
| 17 | Unknown_46.866, 3064, m/z169 | 1.7431 | 1.5167 | 1.3386 | 1.2995 | 1.3504 |
| 18 | Serotonin, x TMS, 39.11, 2470* | 1.7352 | 1.3818 | 1.4892 | 1.4761 | 1.475 |
| 19 | L-Methionine, 1 TMS, 20.27, 1416_putative.2 | 1.7206 | 1.6046 | 1.4578 | 1.4154 | 1.3856 |
| 20 | Unknown_46.848, 3063, m/z169 | 1.705 | 1.4964 | 1.3155 | 1.2779 | 1.3424 |
| 21 | Mix C Unknown 3, 24.71, 1617 | 1.6537 | 1.3595 | 1.2743 | 1.3007 | 1.3123 |
| 22 | Serotonin, x TMS, 39.11, 2470 | 1.6398 | 1.346 | 1.7673 | 1.7197 | 1.7153 |
| 23 | Sugar alcohol, 6 TMS, 30.6, 1915* | 1.6115 | 2.1049 | 1.8296 | 1.7866 | 1.7516 |
| 24 | L-Leucine, 2 TMS, 16.74, 1274 | 1.5988 | 1.3083 | 1.164 | 1.1295 | 1.1066 |
| 25 | Unknown_22.370, 1500, m/z 115 | 1.5933 | 1.3401 | 1.2451 | 1.2235 | 1.1973 |
| 26 | Unknown_23.546, 1558, m/z 292-threonic acid-like | 1.531 | 1.2609 | 1.1703 | 1.1672 | 1.1551 |
| 27 | Unknown_21.778, 1476, m/z234_arabino-hexos-2-ulose-like | 1.5276 | 1.2282 | 1.167 | 1.1653 | 1.2007 |
| 28 | Unknown_40.714, 2581, m/z217.1 | 1.5078 | 1.5118 | 1.3854 | 1.4019 | 1.3849 |
| 29 | Unknown_44.002, 2820, m/z131 | 1.4809 | 1.1772 | 1.4199 | 1.3806 | 1.3759 |
| 30 | Potential artifact | 1.4685 | 1.4697 | 1.5635 | 1.5188 | 1.487 |
|  |  |  |  |  |  |  |

*Additional quantitation calculated from lesser abundant ion, to account for MS detector saturation

#### Table S13. Variable Importance in Projection measures of the top 30 ranked metabolites (sorted by component one) from Partial Least Squares discriminant analysis (PLS-DA) of liver samples, grouping by population.

| # | Metabolite name | Comp. 1 | Comp. 2 | Comp. 3 | Comp. 4 | Comp. 5 |
| --- | --- | --- | --- | --- | --- | --- |
| 1 | D--Turanose, 7 TMS, 42.29, 2702 | 2.7638 | 1.7884 | 1.7221 | 1.6728 | 1.6386 |
| 2 | Unknown_42.354, 2704, m/z217 | 2.5727 | 1.634 | 1.6602 | 1.6266 | 1.5993 |
| 3 | Unknown_40.226, 2546, m/z387-glucose-6-phosphate-like.1 | 2.5477 | 3.1925 | 3.0051 | 2.9097 | 2.8608 |
| 4 | Urea, 2 TMS, 16.14, 1249 | 2.507 | 2.15 | 2.0282 | 2.096 | 2.0503 |
| 5 | Thymine, 2 TMS, 19.94, 1403 | 2.4842 | 1.5841 | 1.4779 | 1.4571 | 1.4269 |
| 6 | Unknown_42.354, 2698, m/z289 | 2.4513 | 1.8533 | 1.7544 | 1.7007 | 1.6698 |
| 7 | Unknown_35.122, 2184, m/z357 | 2.4226 | 1.5247 | 1.5012 | 1.4556 | 1.4242 |
| 8 | artifact.1 | 2.1856 | 1.6494 | 1.6703 | 1.6173 | 1.6493 |
| 9 | Unknown_38.338, 2411, m/z217-uridine-3TMS-like | 2.1631 | 1.4135 | 1.3177 | 1.2882 | 1.2812 |
| 10 | Unknown_28.434, 1803, m/z204 | 2.0673 | 1.3307 | 1.332 | 1.3046 | 1.2811 |
| 11 | L-Lysine, 3 TMS, 26.54, 1712 | 2.0644 | 1.3399 | 1.2649 | 1.2288 | 1.2327 |
| 12 | Unknown_43.018, 2746, m/z361 | 2.025 | 1.5446 | 1.5827 | 1.5336 | 1.5082 |
| 13 | Unknown_42.354, 2698, m/z289.1 | 1.9397 | 1.2875 | 1.2884 | 1.248 | 1.2325 |
| 14 | Myo-Inositol, 6 TMS, 33.38, 2081.1 | 1.8873 | 1.7094 | 1.5921 | 1.554 | 1.5318 |
| 15 | Unknown_37.130, 2142, m/z315-myo-inositol-2-phosphate-like | 1.865 | 1.1949 | 1.1405 | 1.1068 | 1.09 |
| 16 | D-Fructose-6-phosphate 6TMS, MEOX, 36.72, 2300-putative | 1.7514 | 1.252 | 1.1658 | 1.1288 | 1.1084 |
| 17 | Unknown_39.098, 2465, m/z285, m/z285 | 1.7498 | 1.446 | 1.4806 | 1.47 | 1.4381 |
| 18 | Sucrose, 8 TMS, 41.32, 2630.1 | 1.734 | 1.0999 | 1.1264 | 1.1165 | 1.1181 |
| 19 | Sucrose, 8 TMS, 41.32, 2630 | 1.6967 | 1.1554 | 1.1459 | 1.1147 | 1.0904 |
| 20 | Unknown_34.17, 2127, m/z319-galactose-like | 1.6383 | 1.317 | 1.2268 | 1.1879 | 1.1647 |
| 21 | Unknown_31.554, 1971, m/z191-glucose, pentakis-O-TMS-like_Mix A Unknown 11, 31.54, 1971 | 1.6362 | 1.1109 | 1.043 | 1.019 | 0.99684 |
| 22 | a-Ketoglutaric acid, x TMS, 23.95, 1578 | 1.6361 | 1.0579 | 0.98582 | 0.97358 | 0.95636 |
| 23 | beta-Alanine, 3TMS, 20.58, 1429 | 1.6332 | 1.1056 | 1.0311 | 1.0273 | 1.0199 |
| 24 | Unknown_30.49, 1908, m/z 174-dopamine-like | 1.6247 | 1.2899 | 1.2179 | 1.1795 | 1.1537 |
| 25 | Unknown_34.074, 2121, m/z319-galactose-like | 1.5404 | 1.2838 | 1.1963 | 1.1598 | 1.1359 |
| 26 | D-sorbitol m/z_205 | 1.4961 | 1.4887 | 1.479 | 1.4416 | 1.4131 |
| 27 | Cellobiose, x TMS, 42.19, 2962 | 1.4796 | 1.1205 | 1.0433 | 1.0706 | 1.0498 |
| 28 | Unknown_28.986, 1831, m/z218 | 1.4655 | 0.96717 | 0.91146 | 0.89871 | 0.87941 |
| 29 | Unknown_42.674, 2721, m/z361 | 1.4613 | 1.0824 | 1.1053 | 1.1084 | 1.0914 |
| 30 | Unknown_21.930, 1482, m/z 257 | 1.4133 | 1.2137 | 1.1733 | 1.1416 | 1.1166 |
|  |  |  |  |  |  |  |

#### REFERENCES

Grogan, L.F., Mulvenna, J., Gummer, J.P.A., Scheele, B.C., Berger, L., Cashins, S.D., McFadden, M.S., Harlow, P., Hunter, D.A., Trengove, R.D., Skerratt, L.F., 2018. Survival, gene and metabolite responses of Litoria verreauxii alpina frogs to fungal disease chytridiomycosis. Scientific Data 5, 180033.
